# Assessment of self-doped poly (5-nitro-2-orthanilic acid) as a scaling inhibitor to control the precipitation of CaCO_3_ and CaSO_4_ in solution

**DOI:** 10.1038/s41598-022-13564-9

**Published:** 2022-06-13

**Authors:** Hammed H. A. M. Hassan, Dalia E. Abd-El-Khalek, Marwa Abdel Fattah

**Affiliations:** 1 Chemistry Department, Faculty of Science, Alexandria University, P.O. 2, Moharram Beck, Alexandria, Egypt; 2 National Institute of Oceanography and Fisheries, NIOF, Cairo, Egypt; 3 Menoufia Higher Institute of Engineering and Technology MNF-HIET, Menoufia, Egypt

**Keywords:** Engineering, Materials science

## Abstract

Self-doped- and nitro-polyanilines have become a widely used strategy to optimize the electronic and vibratory spectra of polymeric building blocks in various applications. We report the synthesis of poly (5-nitro-2-orthanilic acid) by an aniline-initiated oxidative polymerization reaction. The polymer is characterized by spectroscopic techniques, elemental shapes, cyclic voltammetry, electrical conductivity, and microscopic and thermal measurements. The hydrophilic and hydrophobic nature of the supports provided the formation of amphiphilicity as judged by SEM. Thermogravimetric measurements reveal thermal stability up to 500 °C and glass temperature (T_g_) observed at 240 °C. Electrical conductivity decreases as the temperature rises at the different frequencies used, reflecting the semiconducting nature in the extrinsic range, which is characterized by high carriers and low mobility. The presence of these electron residues causes a decrease in efficiency and increases the thermal conductivity. Dielectric measurements have shown that permittivity decreases gradually at lower levels, mainly due to the transport of charging carriers, resulting in higher performance. The testing of the copolymer as a new scale blocker has resulted in moderate to fairly high performance. This effect is attributed to the change in polymer geometry using intramolecular H-bonding group -SO_3_H and a chain polymer in an aqueous medium.

## Introduction

Scale formation is commonly encountered and poses problems in water boiler coolers and oil well water, which cause a negative impact on operating systems and equipment, reducing heat transfer as an insulating layer and downhole completion equipment^1^. Among the commonly encountered scaling cations in aqueous systems are Ca^2+^ cations, which deposit calcium carbonate and/or calcium sulfate. Sulfate scales are often attributed to the mixing of incompatible sea and formation waters, where the concentrations of calcium ions are high in both^2^. Carbonate scale, on the other hand, is generally attributed to the process of self-scaling, where the loss of carbon dioxide gas from the water to the hydrocarbon phase occurs as pressure falls^3^. The main carbonate scales are calcite CaCO_3_, valaterite CaCO_3_, aragonite CaCO_3_, siderite FeCO_3_, and dolomite CaMg(CO_3_)_2_. Functionalized organic polymers are green scale inhibitors and have shown excellent properties in delaying, reducing, and/or preventing scale deposition^4^. The effectiveness of these inhibitors depends on the chain length of the polymers and thus, the efficiency order in most cases was a low chain- > higher chain length, where the polymeric repeating units were nearly 10–15^5,6^.

The preparation of self-doped polyaniline^7^ has become a widely used strategy and the results clearly indicated that introducing the -SO_3_H (electron-attracting) group on the polyaniline backbone makes them promising building blocks for different directions in applications^8^. In addition, the introduction of –NO_2_ group substitution into polyaniline chains modifies the electronic and vibrational spectra of the resultant polymer^9^. Generally, aniline derivatives containing weak electron-withdrawing groups form a stable free radical during polymerization and the reaction occurs slowly due to some decrease in the electron density on the aniline’ nitrogen atom^10^. However, anilines containing a strong electron-withdrawing substituent, such as –NO_2_, produced an unstable radical intermediate, and thus, polymerization does not occur. To solve this obstacle, a trace amount of aniline as an initiator/promoter improved the reaction rate and yield^11,12^. The ready synthesis poly (nitroaniline-coaniline) in various molar ratios has been used as an effective green step for the removal of toxic byproducts obtained from various dyes and textile industries^13^. Nevertheless, investigations concerning introducing –NO_2_ substituent onto self-doped polyaniline and the expected variation of physicochemical properties in the resultant polymers remain unexplored. In this work, poly (4-nitroaniline-2-sulfonic acid) catalyzed by aniline (10 mol %) was prepared by a persulfate oxidative polymerization procedure and the resultant polymer was characterized by various techniques such as FTIR- and UV–visible spectroscopies, elemental analysis, scanning- and transmission electron microscopy (SEM, TEM), thermal analysis (TGA, DTG, DSC), electrochemical behavior and electrical and dielectric properties. Notably, derivatives based (4-nitroaniline-2-sulfonic acid) were used, among many others, as sensing elements of optical sensors to determine sulfate in water and soil extracts^14,15^. As a continuation of our recent interests^16,17^, the assessment of the prepared poly (4-nitroaniline-2-sulfonic acid), for the first time, as a new scale inhibitor of CaCO_3_ and CaSO_4_ using slandered NACE, electrochemical tests and microscopic examinations was the main objective. The measurements were performed on as-synthesized samples, without the further redoping procedure usually used in the literature.

## Results and discussions

**Figure 1:**
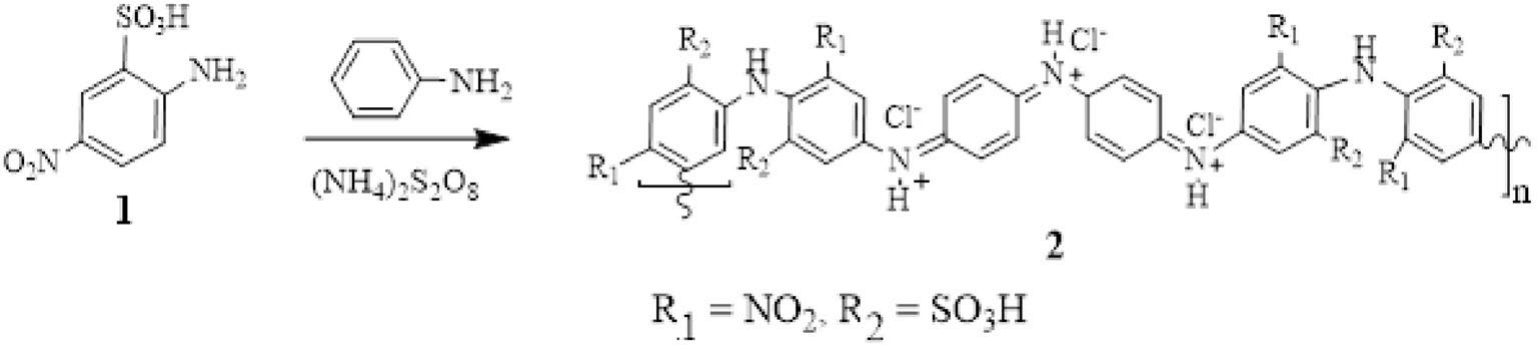
Chemical synthesis of aniline-catalyzed poly (5-nitro-2-orthanilic acid).

**Figure 2:**
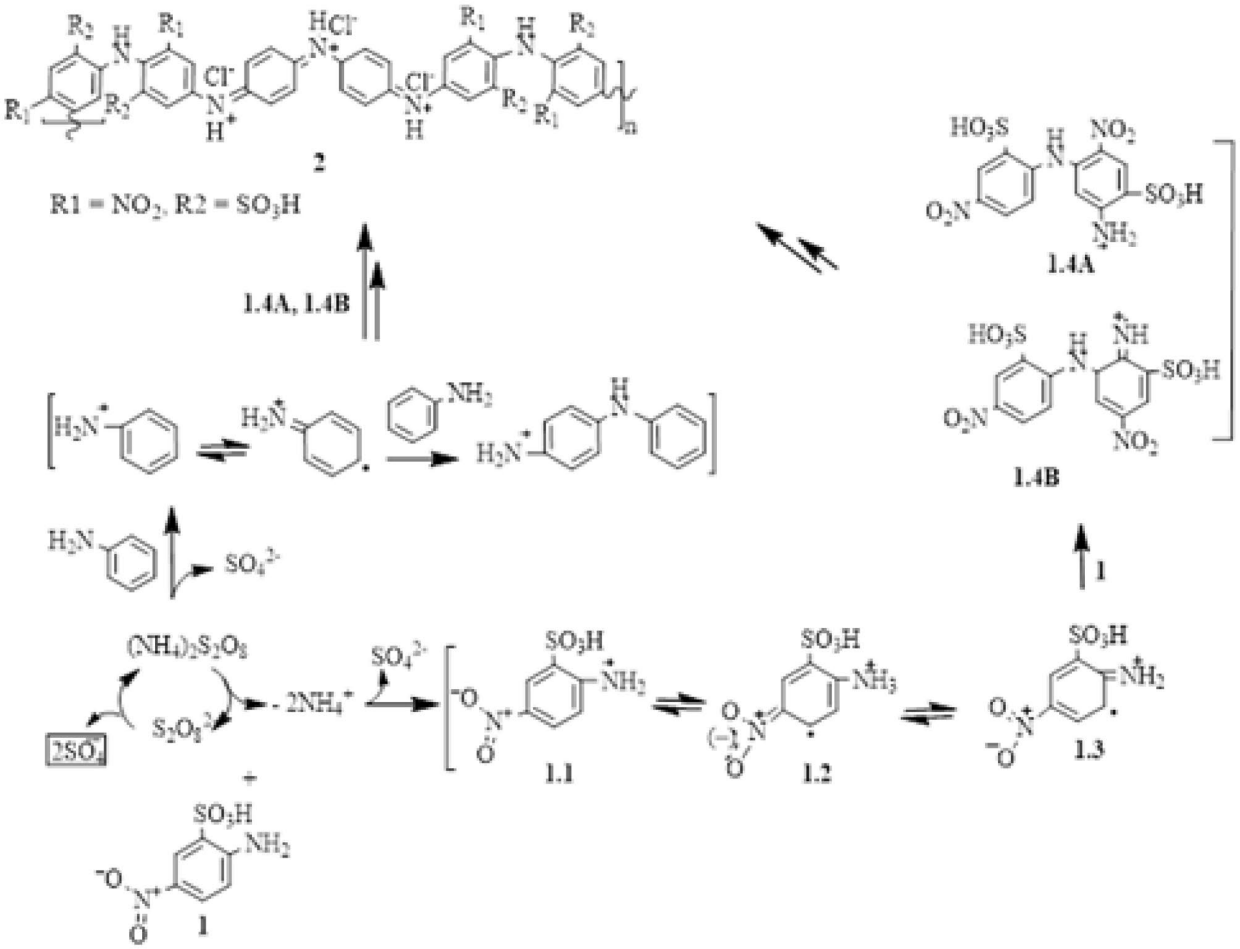
Proposed formation mechanism of polymer **2.**

The first trials to obtain pure poly (5-nitro-orthanilic acid) **2** failed and even if the reactants were stirred for 48 h produced only a yellow precipitate contained an unreacted and probably dimeric mixture. Polymerization of aniline-2-sulfonic acid (orthanilic acid) was chemically achieved only at high pressure^18^ to give the targeted polyorthanilic acid. Typical oxidative polymerization of orthanilic acid is not possible due to the −I and steric hindrance effects of the –SO_3_H group^19^. In substrate **1** used in this investigation the presence of a strong electron withdrawal effect exerted by the substituents –SO_3_H and –NO_2_ group inhibits the first initiating oxidation step. Therefore, using a small amount of aniline to initiate the polymerization reaction was our alternative^20^. Targeted poly (5-nitro-2-orthanilic acid) **2** was chemically prepared, according to the standard procedure described in the IUPAC technical report^21^, using 10 mol % aniline and ammonium persulfate as an oxidizing agent from commercial 5-nitro-orthanilic acid **1** in low pH 1.5 aqueous HCl media, Fig. 1. The percent yield (52%, η_inh_ = 0.10) was calculated by using the formula^22^: [% Polymer yield = (Weight of polymer / Weight of substrate 1) × 100]. The reaction rate depends mainly on the reactivity of the reactant substrate as well as any hindrance to reaction propagation which greatly affects the yield. The –SO_3_H and –NO_2_ substituents are present at the ortho and para positions with respect to the –NH_2_ group, respectively, and would electronically direct the propagation taking place solely at the C3 position. During the propagation step, the ortho –SO_3_H group would reduce the nucleophilicity of the –NH_2_ group and exert a steric hindrance, nevertheless, further head-to-tail oxidative polymerization can be expected by the unsubstituted comonomer aniline and thereby to achieve a fair polymerization yield. The postulated reaction mechanism is shown in Fig. 2.

**Figure 3:**
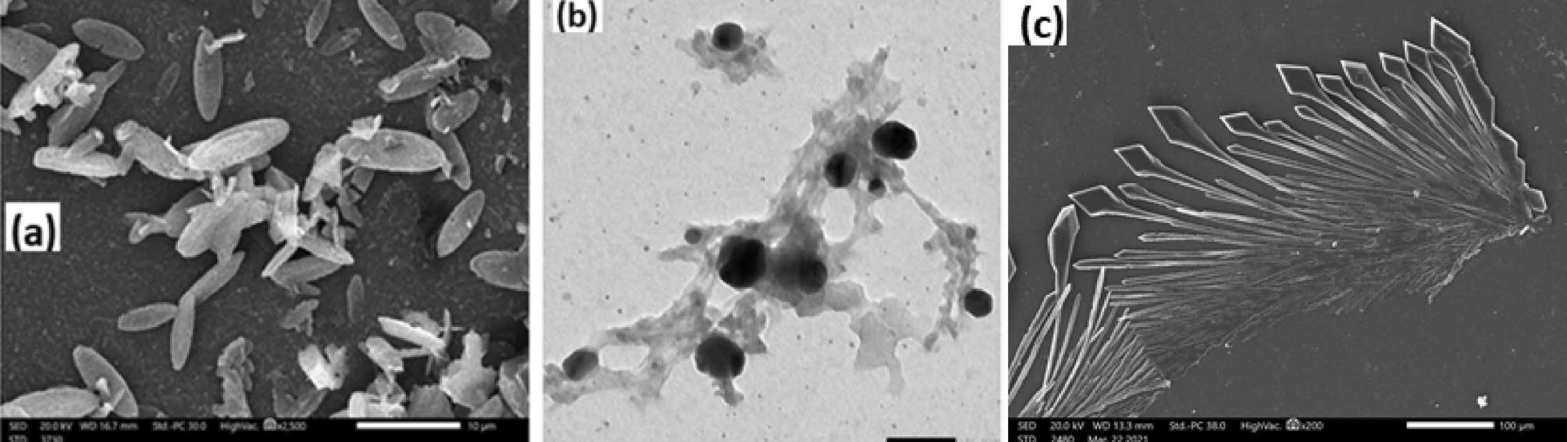
(**a**) SEM and (**b**) TEM images of the prepared Poly (5-nitro-orthanilic acid) 2 initiated by aniline. Figure 1(c) SEM image of uncatalyzed polymerization product.

The morphology of synthesized polymer **2** investigated by SEM; (Fig. 3a) revealed that the polymer has a regular flower-leaf-like microstructure. However, the TEM image, Fig. 3b, exhibited particle microaggregates and clearly indicated the presence of both unsubstituted aniline units (dark nanospheres) and substituted polymeric units (light gray microaggregates). For comparison purposes, an SEM image of the product obtained from uncatalyzed polymerization trials is shown in Fig. 3c. The morphology and the particle sizes are attributed to the nature of the substituent and thus the mechanism of the monomer unit interactions^23,24^. The presence of a monomer hydrophilic –SO_3_H group and a hydrophobic –NO_2_ on one aromatic ring gives the structure amphiphilicity or self-assembly nature, which are surrounded by the aromatic ring forming micelles^25^ and the aggregation of such small micelles produces submicron groups that are templates for such morphology.

## Elemental analysis

The elemental compositions of polymer **2** were: C, 30.35; H, 5.012; N, 17.15; C/N ratio 1.76 [Calc. for C_19_H_36_N_9_S_2_Cl_2_: (750); C, 30.31; H, 5.05; N, 16.82; S, 8.55; Cl, 9.46; C/N ratio 1.80]. Analysis of sulfur contents showed 11.40% and therefore the S/N ratio = 0.66 indicating a high content of substituted units in the resulting polymer backbone. The data for C and N are in good agreement with the chemical formula of a modified polyaniline backbone. The total amounts of carbon, hydrogen and nitrogen in the resulting polymer were 90.94%, indicating the contamination of the polymer with chlorine and/or ammonia as speculated doped emeraldine salt forms^26^. This could be attributed to the presence of ammonium hydrogen sulfate, which was not able to fully wash out during the working up of the product. The ratio of C/N was 1.76 (Calc. 1.75) and agrees with the theoretically predicted values for their analogues^27^. Notably, the elemental data also indicated that ammonium species were incorporated into the polymer product during the polymerization.

## Infrared spectroscopy

The FTIR spectrum shows absorption at υ 3359–3425 cm^−1^ that corresponds to the NH_vib_ bond, and the peak in the region of υ 2925–3000 cm^−1^ is due to the vibrations of the C–H_arom_ bonds. The band at 2707 cm^−1^ suggests the existence of ammonium salts^27^. Characteristic polymer structure peaks were found at υ 1500 and 1571 cm^−1^ corresponding to the vibrations of the benzenoid and quinoid moieties and their intensity ratio was 1.3, confirming the rapid equilibrium between the two forms^28,29^. The relative intensity of the quinoid/benzenoid bands reflects not only the degree of oxidation of the polymer chain^30^, but also confirms the formation of bipolaron in the initial oxidation stage. The absorption bands at υ 1321 cm^−1^ and υ 1254 cm^−1^ correspond to the C–N and C–N^+^ stretching vibrations, respectively. The peak observed at υ 1221 cm^−1^ corresponds to the N=O symmetric stretching vibration due to NO_2_ groups. The asymmetric stretching band of the N=O group was not observed and could be buried under C–N absorption. The presence of a peak at υ 1074–1131 cm^−1^ indicates the doped state of the polymer (–N^+^/ N^+•^). The absorption at υ 828 cm^−1^ corresponds to the bending vibrations of the C–H in plane of the aromatic ring. The S–O stretching band appeared at υ 748 cm^−l^, and the peak at υ 620 cm^−1^ was attributed to the C–S stretching vibration^31^.

## Ultraviolet-Vis absorption spectroscopy

The bandgap energy is calculated from the equation: ΔE = hc/λ, where ΔE is the bandgap energy (eV), h = 6.625 × 10–34 JS, c = 3 × 108 m/s, and λ is the wavelength. The electronic spectra of the substituted polymer **2** show absorption bands at 255 nm, and 380 nm and their bandgap energies were 4.86 eV and 3.26 eV, respectively. The observed absorptions are assigned to the bandgap and the absorption data show bathochromic shifts. The increase in bandgap occurred because of the increasing torsion angle between the C–N–C plane and the plane of the benzene ring, due to the electronic nature of the substituent and thus affecting the conjugation degree. Due to random substitution of the monomers of such polymerization, the substituent causes entangling of polymer chains that disrupt the conjugation length which results in a hypsochromic shift of the π–π* transition at 255 nm and the polaron-π* transition at 380 nm.

## Cyclic voltammetry

**Figure 4:**
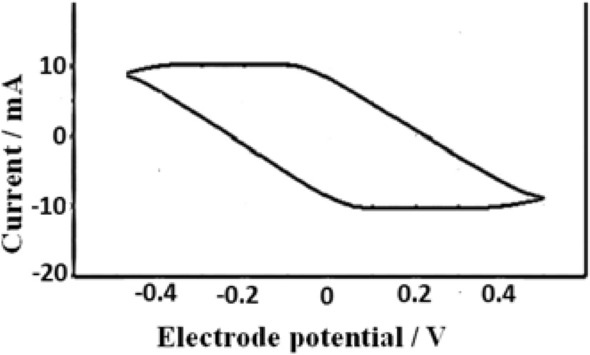
Cyclic voltammogram of the prepared substituted polymer 2. Scan rate of 100 mV s^−1^.

The electrochemical study of substituted copolymer **2** was conducted by measuring the cyclic voltammogram; (Fig. 4). Polyaniline itself is known to be a redox polymer; therefore, incorporation of substituent into its structure affects the oxidation and reduction potentials observed on the cyclic voltammetry that are related to a change in its redox form. The cyclic voltammetry maxima correspond to transitions between the oxidation forms.

**Figure 5:**
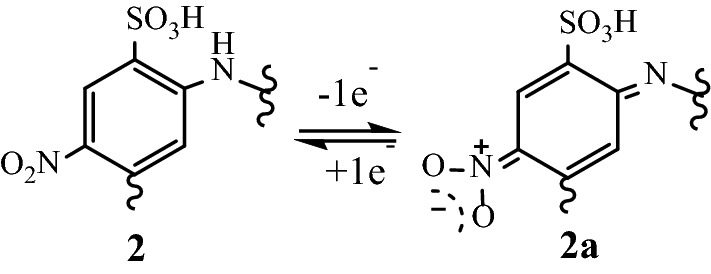
Reduced and resonating oxidized structures of the functionalized monomeric moiety in the copolymer **2.**

Copolymer **2** was subjected to electrochemical tests to study the electron transfer behavior. The experiment (one cycle) was conducted at a concentration of 5 mg in 20 ml DMSO solution. The voltammogram wave shape showed only one broad cathodic peak at 0.056 V, assumed for the reduction of the quinoid structure in the polymer chain, and one broad anodic peak at −0.090 V, respectively. This electrochemical behavior could be attributed to the presence of electron-withdrawing, –SO_3_H and –NO_2_ groups which can be oxidized to quinone and vice versa at different potentials^32^, Fig. 5. The cyclic voltammogram of copolymer **2** exhibited good reversibility.

## Thermal analysis

**Figure 6:**
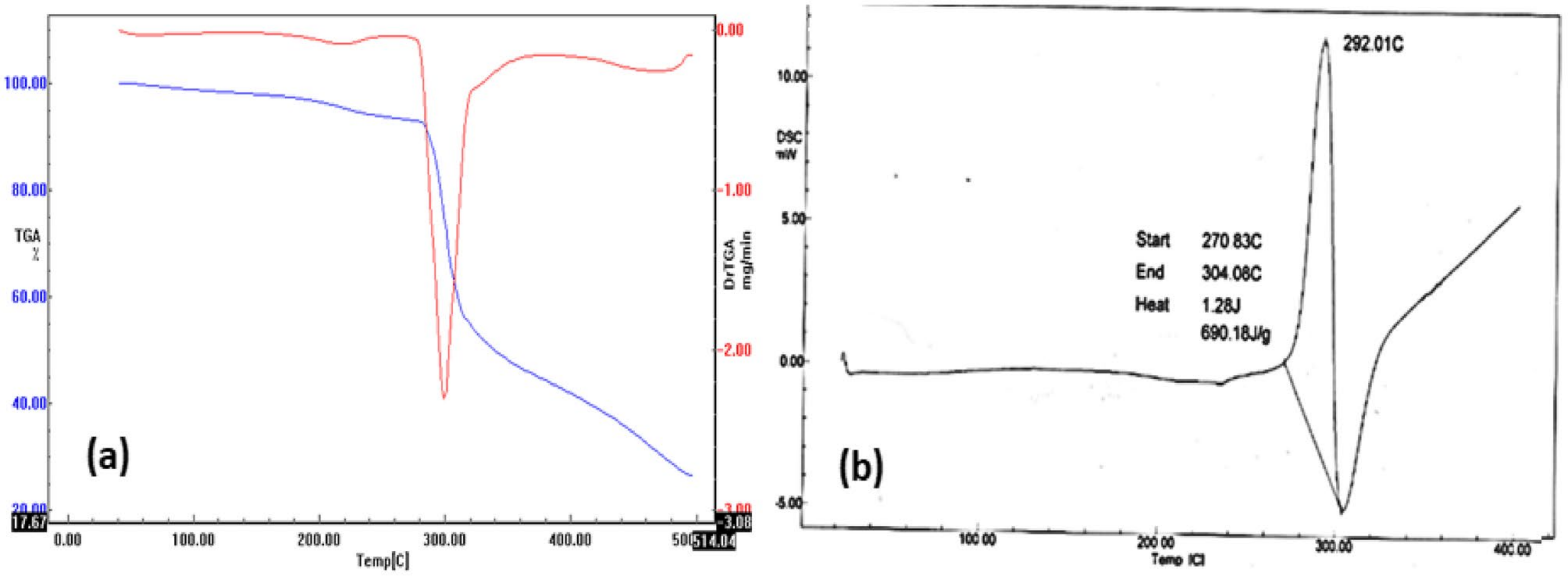
(**a**) TG-DTG and (**b**) DSC thermograms of the substituted polymer **2.**

TG-DTG and DSC curves for the substituted polymer **2** are shown in Fig. 6. The TG thermal degradation curve, Fig. 4a, exhibited an interesting model of the stability of the polymer and its subsequent weight-losses could be divided into four steps. The first minor peak from rt to 198 °C could be attributed to the loss of absorbed water. The second minor weight loss from 197 to 279 °C is attributed to the loss of acid dopant from the polymeric matrix. The third weight loss (major peak) from 281 to 346 °C (strong differential thermogravimetric endothermic peak at 292 °C) 345–466 °C is likely due to decomposition and/or elimination processes of the side-chain substituents. The oxidative thermal decomposition of polymer backbones was suggested above 500 °C for the remaining polymeric residues (31%) as the final weight loss. In the DSC curve (Fig. 4b), the polymer shows a strong exothermic peak with an energy of 690.18 J/g at 292 °C, as well as a strong endothermic peak at 305 °C, which corresponds to decomposition or elimination processes of the side-chain substituents and the subsequent morphological change in the polymer^33^. The glass temperature (T_g_) of the polymer was observed at 240 °C.

## Electrical conductivity

### Dielectric measurements

**Figure 7:**
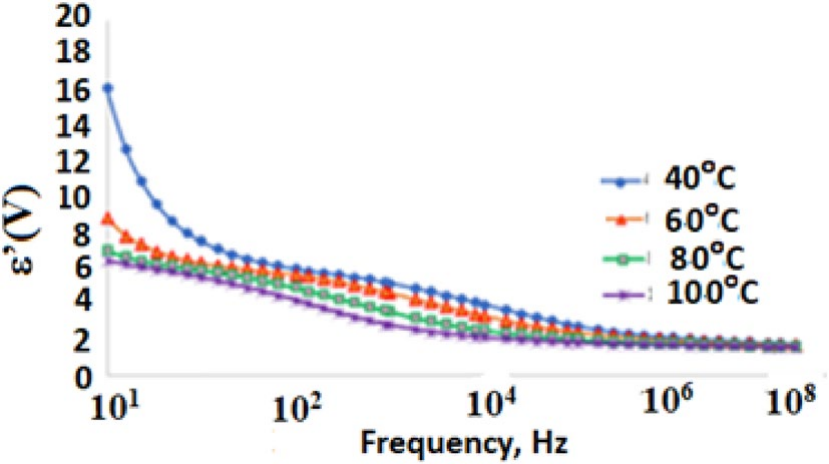
Effect of temperature on permittivity at different frequency for the polymer **2.**

The dielectric measurements carried out using a high analyser technique could probe molecular fluctuations and charge transport over broad frequency and temperature ranges. The dielectric spectrum is separated by molecular dynamics at the molecular scale, i.e., charge carrier transport, which is reflected in conductivity mechanisms. The permittivity (ε*) is the measure of resistance that is developed upon generating an electric field in a particular substance. The permittivity (ε*) was determined at five-spot frequency points and depicted against temperatures in the range of 40 °C to 100 °C for the substituted polymer **2** (Fig. 7). In general, two distinguished trends of the real part of the complex permittivity ε′ vs frequency can be assigned. The lower frequency range (0.1 Hz–10 kHz) shows a gradual decrease in ε′ through six orders of magnitudes, mainly due to the charge carrier transport causing the expected high conductivity. At a higher range of frequencies (10 kHz − 20 MHz), a slight effect was observed for frequency on decreasing the permittivity. This effect agrees with the fact that at higher frequencies, the contribution of the alteration of all kinds of polarizations lags the frequency of the applied external electric field^34^.

**Figure 8:**
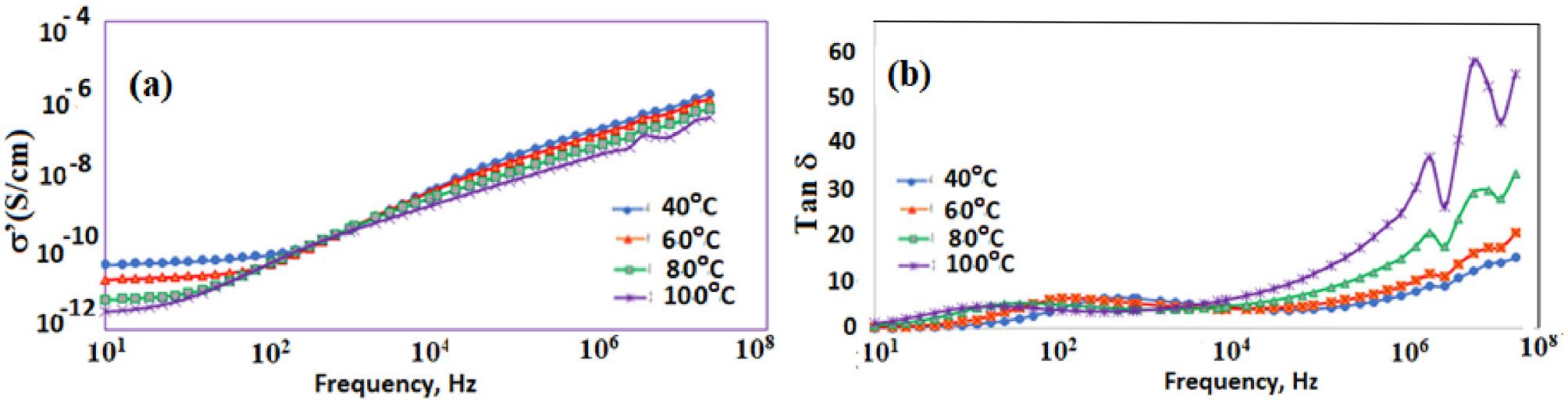
(**a**) Conductivity (σ′) vs frequency- and (**b**) Dissipation factor (tan δ) vs frequency for polymer **2** at temperature range 40–100 °C.

The variation in conductivity σ’ vs frequency (DC-conductivity) and the dissipation factor tan δ vs frequency at a temperature rang (40–100 °C) are illustrated graphically in Fig. 8a and b for polymer **2**, respectively. Figures show a clear sharp peak followed by a small shoulder at lower limit points of frequency. At higher frequencies, it follows the power law: [σ’ (ω) = Aω^5^], in which A is constant and s characterizes the rate of change of AC-conductivity with increasing frequency. The intermediate range of frequency shows a plateau-like behavior that represents the dc-conductivity, σdc. It varies slightly between 1 and 2 mS/cm by the variation of the irradiation dose, which confirms the fact that the contribution of the conductivity plays the main role of the permittivity values at the lower frequencies. The accumulation of charge carriers is the origin of electrode polarization, a ubiquitous phenomenon that takes place at the interface between a metallic and an ionic conductor and thus; increases the net dielectric response of the sample cell by many orders of magnitude. Since Coulombic interactions take place here, ion mobility is drastically slowed down at the interfaces.

**Figure 9:**
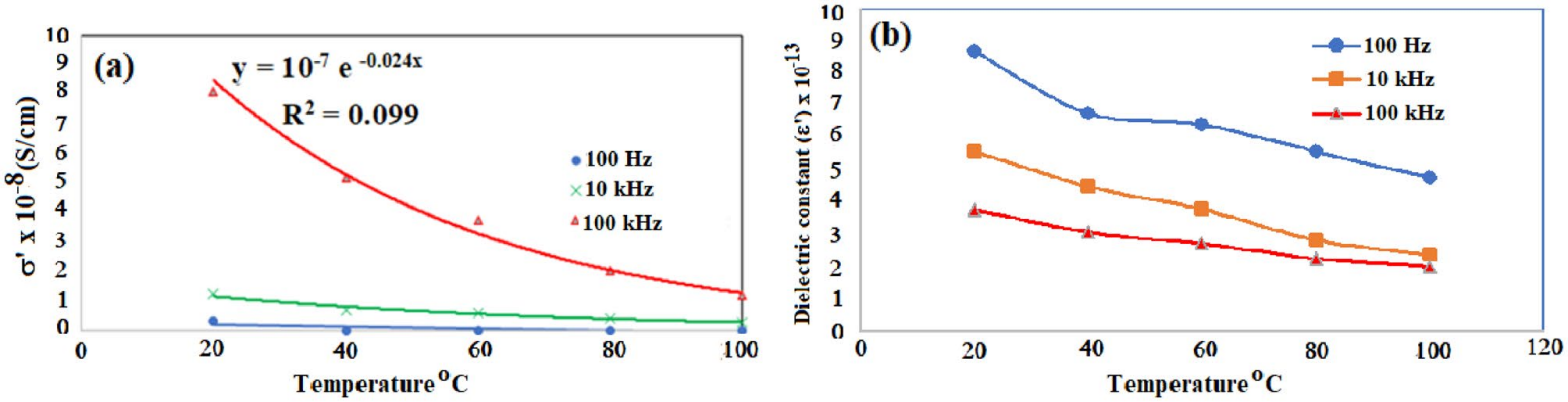
The variation of conductivity σ′ vs Temperature for polymer **2** at 100 Hz, 10 kHz and 100 kHz.

Figure 9 illustrates the effect of temperature on the conductivity σ’ and dielectric constant ε’ plotted at different frequencies (i.e.,100 Hz, 10, and 100 kHz) for polymer **2**. In Fig. 9a, as the temperature increases, the conductivity decreases at different applied frequencies. This behavior is indicative of a semiconducting nature in the extrinsic range, which is characterized by high carriers and low mobility. The main two factors affecting the value of conductivity are the density of the charge carriers, n, and their mobility, μ. The charge density and mobility as a function of temperature, and the effect of the change in charge density only plays a limited role in the variation in the DC conductivity. This can be attributed to the fluctuation dynamics of the accumulated charge carriers at the interface. These fluctuation dynamics seem to be the reason for the asymmetric wing of the σ″ peak at lower frequencies which is accompanied by a shoulder in the tan δ and deviation from the linearity of the conductivity decrease shown in Fig. 9a ^35^. The low conductivity of copolymer **2** could be explained by both the reduced conjugation length in distorted chains and the decrease in interchain charge transport induced by bulky SO_3_H groups, and the electrostatic interactions between the sulfonate functional groups and main-chain cationic nitrogen or amine hydrogens. The study of the dielectric constant as a function of temperature and frequency is one of the most convenient methods of studying the molecular orientation behavior and associated relaxation mechanism of polymer structures^36^. In Fig. 9b, the dielectric constant decreases with increasing frequencies. At higher frequencies, the value of the dielectric constant nearly steadily decreases. However, at low frequencies, the dielectric constant decreases in the (20–40 °C) temperature range, remains stable in the (40–60 °C) range and then continues to decrease. This nature is not observed at higher frequencies. The low-temperature dielectric dispersion is attributed to the dielectric response of the side groups which are more mobile or the small displacement of the dipoles near the frozen-in position, which is known as the secondary dispersion region or ß-relaxation^37^.

## Assessment of copolymer 2 as a precipitation inhibitor of CaCO_3_ and CaSO_4_ in solution

### Effect of copolymer 2 on CaSO_4_ precipitation

**Figure 10:**
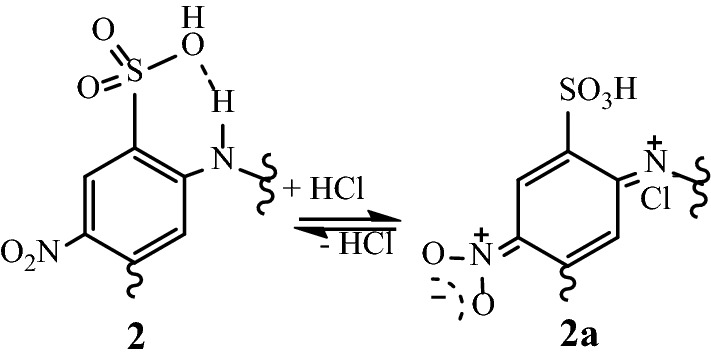
Proposed intramolecular interaction of the ortho-sulfonic group and the polymeric chain.

To examine the synthesized copolymer **2** as a new scale inhibitor of CaSO_4_, the free calcium ions concentration was determined according to the NACE test in the absence and presence of different polymer concentrations (ppm), and the results are compiled in Table 1. The concentration of free Ca^2+^ ions slightly increased with increasing polymer concentration, indicating inhibition of the calcium sulfate precipitation process. In the literature, the inhibition of the CaSO_4_ scale by inhibitor-containing sulfonate groups is usually explained by the interaction of calcium cations present in solution with sulfonate groups through Ca….SO_3_ interaction and thereby blocks crystal growth^16^. In general, while the M–O interaction is weak in a single sulfonate moiety, each oxygen atom has the potential to bridge more than one metal center, and typically, the oxygen atoms of an SO^3−^ moiety will bridge a maximum to two metal ions^38^. The limited CaSO_4_ inhibition efficiency by polymer **2** is attributed, most likely, to a change in polymer geometry because of the expected intramolecular hydrogen bonding interaction of the *ortho*-sulfonic group and the polymer chain in an aqueous medium; (Fig. 10) and thus retards the speculated calcium-sulfonate interaction.

**Table 1:** Concentration of free Ca^2+^ ion in brine solution in the absence and presence of different concentrations of polymer **2** and their percent of inhibition (C_b_ = 1221.22 ppm and C_a_ = 800.8 ppm).

Polymer concentration (ppm)	Ca^2+^ ppm	% Inhibition
10	900.90	24
50	920.92	29
100	960.96	38

## Effect of copolymer 2 on CaCO_3_ precipitation

### Chronoamperometry measurements

**Figure 11:**
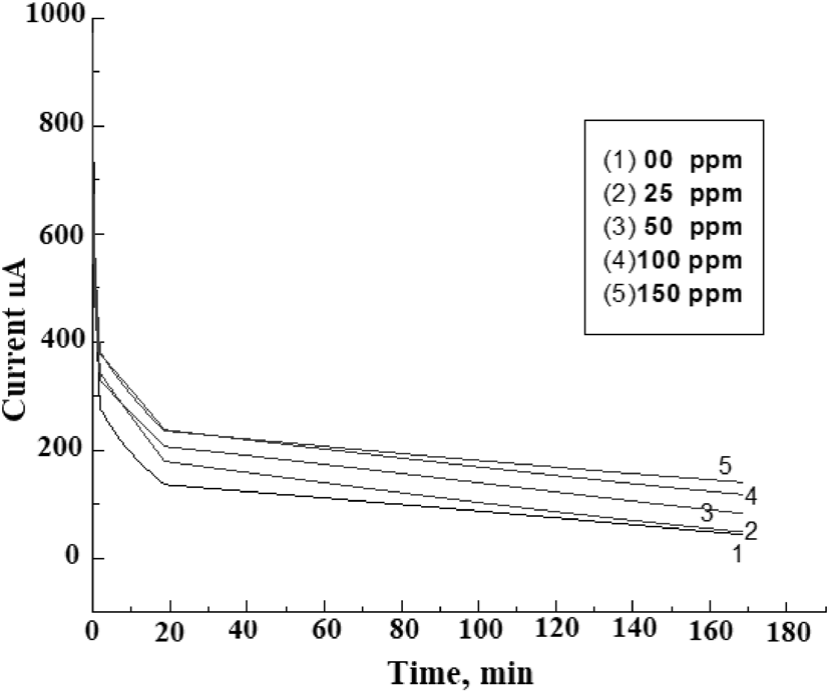
Chronoamperometry curves for polarized steel electrode in CaCl_2_ brine solution in the absence and presence of different concentrations of studied polymer at –1 V vs SCE and 40 °C.

**Figure 12:**
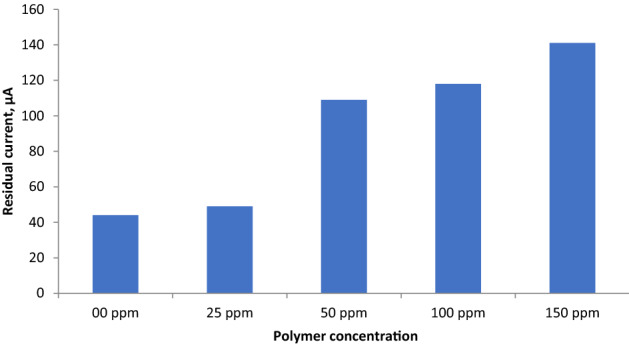
Variation of residual current for steel electrode after 3 h of polarization in CaCl_2_ brine solution in in the absence and presence of different concentrations of studied polymer at –1 V vs SCE and 40 °C.

The buildup of the calcium carbonate layer on the metal surface is illustrated in Fig. 11. As shown, the chronoamperometry curve of the polarized steel electrode in the absence of the polymer decreases sharply, indicating to the fast formation of CaCO_3_ crystals that occupy parts of the steel surface and consequently decrease the current density. On the other hand, the addition of an inhibitor to the brine solution delays the scaling process and increases the residual current on the steel surface. Increasing the concentration of the polymer increases the residual current values for the steel electrode after polarization from 44 μA for a blank solution to 140 μA in the presence of 150 ppm of the polymer; (Fig. 12). Usually, the current density is inversely proportional to the quantity of CaCO_3_ scales formed on the metal surface. Thus, the observed inhibition order indicated that the presence of copolymer **2** significantly inhibits carbonate scale formation.

### Electrochemical impedance measurements

**Figure 13:**
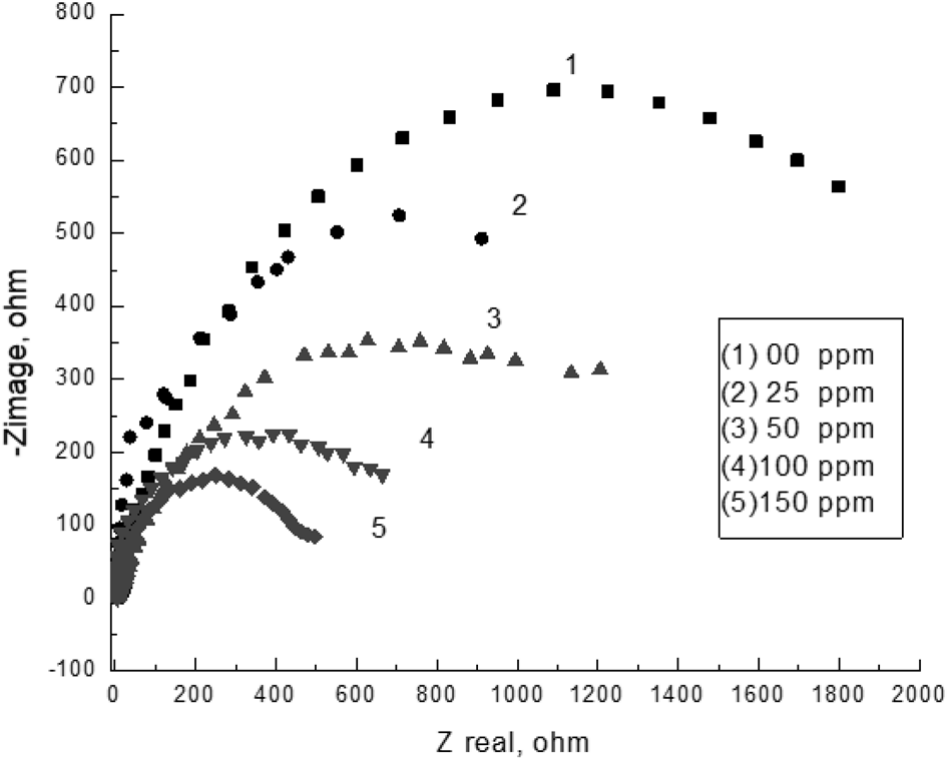
Impedance spectra of polarized steel in brine solution in the absence and presence of different concentrations of the copolymer **2** at 40 °C.

**Figure 14:**
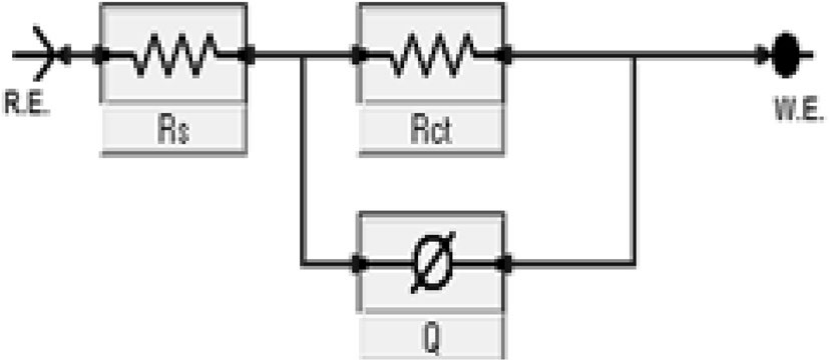
Schematic for the equivalent circuit models used to determine the impedance Parameters for scale process in the CaCl_2_ brine solution. (R_s_; the solution resistance, R_ct_; the charge transfer resistance, Q; is associated to the double-layer capacitance).

**Figure 15:**
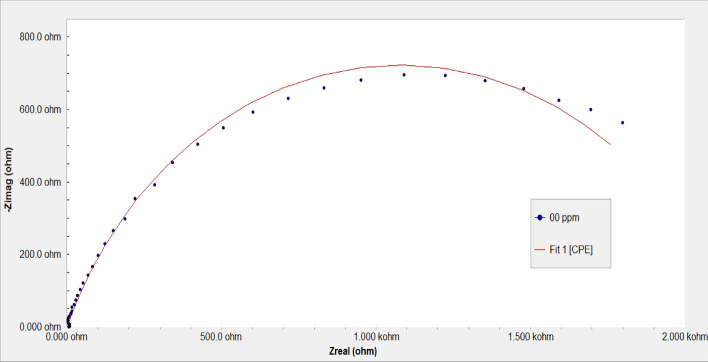
Impedance spectra of polarized steel in brine solution with its fitting curves and equivalent used circuits in the absence and presence of scale inhibitors.

**Figure 16:**
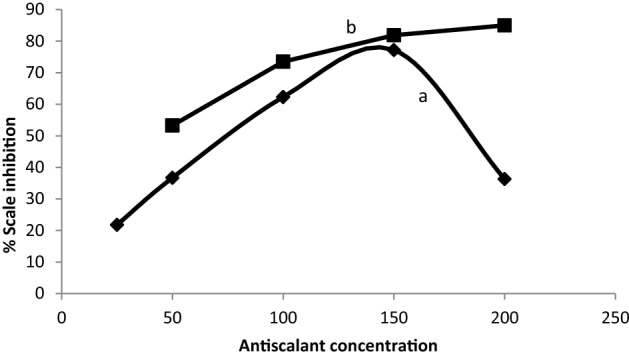
Variation of % CaCO_3_ inhibition with different concentration of: (**a**) Copolymer 2 (**b**) Oxidation product of substrate 1 without addition of aniline initiator.

Figure 13 represents Nyquist plots for steel after cathodic polarization in a brine solution in the absence and presence of different concentrations of copolymer **2**. The impedance spectra of the polymer show a typical feature of depressed semicircles followed by a low-frequency tail. The size of distorted semicircles decreases in the presence of the polymer due to the behavior of the double layer that led to a decrease in the charge transfer resistance due to the reduction of the insulation layer of the scale^16^. The equivalent circuit; (Fig. 14), that was used to fit the experimental data of impedance plots for the scale formation processes in brine solution is illustrated in Figs. 15 and 16.

Table 2 shows the computer fit results of the impedance spectra obtained for the steel electrode that was cathodically polarized in the CaCl_2_ brine solution containing different concentrations of the polymer after 3 h. The percentage of scale inhibition could be determined from the following equation: % scale inhibition = [(Rct_o_ − Rct_i_) / Rct_o_] × 100;^23^ where Rct_o_ and Rct_i_ are charge transfer resistances after polarizing the steel electrode at −1 V (vs. SCE) in a scaling environment for 3 h in the absence and presence of the polymer, respectively. The results indicated that increasing the concentration up to 150 ppm resulted in a decrease in the charge transfer resistance Rct, which is inversely proportional to the formation of scale. Additionally, the presence of the polymer increased the nonideal film capacitance values, Q, due to the formation of the double layer which reduced the formation of the insulating layer on the metal surface. Further increasing the polymer concentration decreases the nonideal film capacitance because of an increase in the thickness of the electrical double layer associated with the presence of polymer molecules which increases the charge density near the metal surface^39^ and decreases the efficiency of the polymer due to the steric effect of the high molecular weight polymer.

**Table 2:** Computer fit results (± error) of the impedance spectra obtained for the steel electrode that was cathodically polarized in CaCl_2_ brine solution containing different concentrations of copolymer **2** after 3 h.

Conc. (ppm)	R_s_ (Ohm cm^2^)	Q (µF)	n	R_ct_ (Ohm cm^2^)	% Inhibition
0	5.03 ± 0.04	209.3 ± 1.15	0.79	2017 ± 23.5	–
25	17.0 ± 0.14	60.13 ± 0.77	0.78	1577 ± 18.4	21.8
50	6.8 ± 0.06	182 ± 2.8	0.68	1276 ± 14.2	36.7
100	1.17 ± 0.039	358.2 ± 2.3	0.79	761.9 ± 11.3	62.3
150	29.7 ± 0.028	361 ± 2.2	0.87	460.3 ± 4.2	77.2
200	8.0 ± 0.048	307 ± 1.9	0.75	1285 ± 14.9	36.3

**Figure 17:**
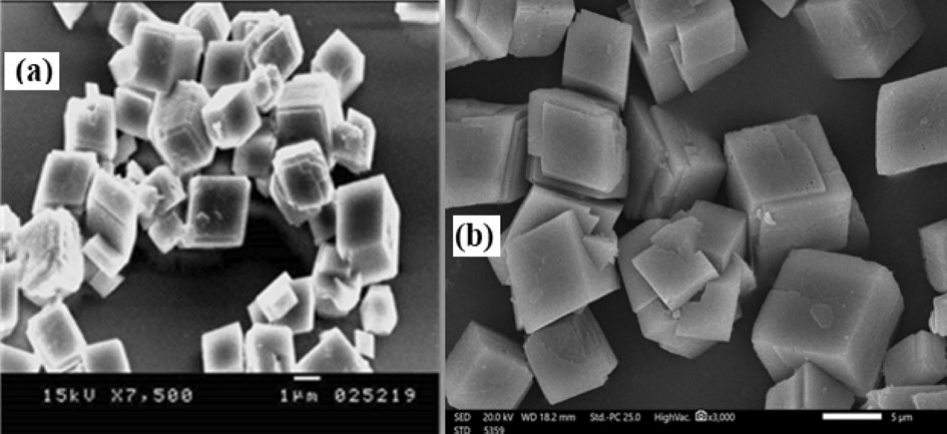
SEM images of CaCO_3_ crystals: (**a**) in absence, and (**b**) in presence of copolymer **2**.

SEM images of CaCO_3_ crystals after direct precipitation are displayed in Fig. 17. The cubic structure of calcium carbonate crystals is observed in the absence of antiscalants. A low degree of crystal modification was observed in the presence of the polymer which indicates that the inhibitory effect is attributed to a change in polymer geometry because of the expected intramolecular hydrogen bonding interaction of the ortho-sulfonic group and the polymer chain in an aqueous medium and thus retards the speculated calcium-sulfonate interaction which facilitates the attachment of polymer molecules on the metal surface and hinders crystal attachment.

## Materials and methods

### Chemicals

Commercial aniline (AlGomhoria Chemicals Co.), 5-nitro-2-aminobenzenesulfonic acid (Aldrich), ammonium persulfate, double distilled water, analytical reagent-grade NaCl, NaHCO_3_, Na_2_SO_4_ and CaCl_2_ (Al-Gomhorya Chemicals Co., Egypt) were used without further purification.

### Measurements

Infrared spectra (IR, KBr pellets; 3 mm thickness) were recorded on a Perkin-Elmer Infrared Spectrophotometer (FTIR 1650). All spectra were recorded within the wavenumber range of 4000–600 cm^−1^ at 25 °C. Absorption spectra were measured with a UV 500 UV–Vis spectrometer at room temperature (rt) in DMSO with a polymer concentration of 2 mg/10 mL. Elemental analysis of the as-synthesized copolymer was performed at the Microanalytical Unit, Cairo University. The sulfur content was determined by the ASTM-D1552 technique at the Middle East Oil Refinery Company (Midor), Alexandria, Egypt. Inherent viscosities (η_inh_) were measured at a concentration of 0.5 g/dL in H_2_SO_4_ at 30 °C by using an Ubbelohde viscometer. Thermogravimetric (TG) and differential thermogravimetric (DTG) analyses were carried out in the temperature range from 20 to 400 °C in a steam of nitrogen atmosphere by a Shimadzu DTG 60H thermal analyser. The experimental conditions were platinum crucible, nitrogen atmosphere with a 30 mL/min flow rate and a heating rate of 10 C/min. Differential scanning calorimetry (DSC-TGA) analyses were carried out using SDT-Q600-V20.5-Build-15 at the microanalytical unit, Cairo University. Cyclic voltammetry was performed using an eDAQ system (www.eDAQ.com), consisting of an E190 potentiostat connected to an e-corder that was inputted to eChem software (running on a PC using Microsoft Windows 10). The working electrode was a 3 mm diameter glassy carbon electrode; the reference electrode was Ag/AgCl; and the auxiliary electrode was a 0.25 mm diameter Pt wire. The applied potentials ranged from −500 to + 500 mV, and the scan rate was 100 mV s^−1^. The volume of the voltammetric cell was approximately 15 ml. The dielectric measurements of the polymeric materials were carried out using a high-resolution Alpha analyser (Novo Control, Hundsangen, Germany) in parallel plate geometry over a frequency range from 10^−1^ Hz to 10^7^ Hz at different temperatures. In this geometry, the sample cell consists of two gold-coated brass disk electrodes. The upper one is 10 mm and the lower one is 20 mm in diameter. The pressed sample with a 12 mm diameter was sandwiched between the two electrodes. The applied voltage was kept constant at 0.2 V to avoid any nonlinear effects. The empty sample capacitor is used as a reference to eliminate the additional contributions of the cables and the measurement cell. The temperature of the sample is controlled by a Quattro Novo control cryo-system with temperature stability better than 0.2 K, as described in references^40–42^. The polymer powder was pressed to form discs with diameters of 10 mm and thicknesses of 1 mm. Silver electrodes were deposited on both sides of the sample surface by thermal evaporation and two copper wires were fixed on the sample using conducting silver paint. The morphologies of the polymers were observed by scanning electron microscope (SEM) (JEOL-JSMIT 200) and transmission electron microscopy (TEM) (JEOL-JTM-1400 plus), at the E-Microscope Unit, Faculty of Science, Alexandria University. The samples were sonicated in deionized water for 5 min, deposited onto carbon-coated copper mesh and allowed to air-dry before examination.

### Synthesis of poly (5-nitro-2-orthanilic acid) 2

5-Nitro-2-orthanilic acid **1** (5 g, 22.93 mmol) and aniline (0.5 g, 5.37 mmol; 10 mol % of **1**) were dissolved in aqueous 1 M HCl (100 ml), and a solution of ammonium persulfate (6.53 g, 28.65 mmol, 1.25 ×) dissolved in water (50 ml) was subsequently added over a period of 30 min. The mixture was mechanically stirred for 24 h at rt and the color change pattern during polymerization from yellow to light green; and then dark green to brownish black was observed from t = 10 min to 24 h. Polymerization was stopped by the addition of methanol (50 ml). The resulting precipitate was subsequently washed with water, aqueous 1 M HCl, water and acetone to remove the unreacted starting materials and short oligomers. Finally, the deep brown precipitate was dried in a vacuum oven at 50 °C. Yield: 2.6 g (52%). IR (cm^−1^, υ): 3434, 3346, 3247, 1789, 1642, 1606, 1571, 1500, 1481, 1406, 1321, 1254, 1221, 1131, 1074, 1018, 918, 828, 748, 648, 620, 573, 516. UV–vis (λ_max_ nm): 255, 380. Calc. for C_19_H_36_N_9_S_2_Cl_2_: (750); C, 30.31; H, 5.05; N, 16.82; S, 8.55; C/N ratio 1.80; Found: C, 30.35; H, 5.012; N, 17.15; C/N ratio 1.76. Sulfur content found: 11.40; S/N ratio 0.66.

## Determination of the scale inhibitor rate

### NACE test for CaSO_4_ scaling

Following the reported method described previously^43,44^, calcium brine: 7.5 g/L NaCl + 11.0 g/L CaCl_2_.2H_2_O and sulfate brine: 7.5 g/L NaCl + 10.66 g/L NaSO_4_, 50 ml of each brine solution was connected in the test cell with different concentrations of sodium alginate and chitosan. Testing cells were placed in the water bath set at 71 °C for 72 h. Then, the concentration level of calcium ions was determined in the solution by titration with EDTA and Murexide indicator. The scale inhibitor percent was calculated following the equation: % inhibition = 100 × (C_a_−C_b_)/(C_c_−C_b_), where C_a_ = Ca^2+^ concentration in the treated sample after precipitation, C_b_ = Ca^2+^ concentration in the blank after precipitation and C_c_ = Ca^2+^ concentration in the blank before precipitation.

## Electrochemical test for CaCO_3_ scaling

Monitoring the buildup of calcium carbonate layers on the metal surface was studied by electrochemical methods including chronoamperometry and electrochemical impedance spectroscopy^16^.

### Chronoamperometry test

The cathodic polarization of the steel electrode initializes the scaling process by forcing few nuclei of CaCO_3_ to be precipitated on the steel surface according to the following equations:

1 $${\text{O}}_{2} + 2{\text{H}}_{2} {\text{O}} + 4e^{ - } \to 4{\text{OH}}^{ - }$$

2 $${\text{Ca}}^{2 + } + {\text{HCO}}_{3}^{ - } + {\text{OH}}^{ - } \to {\text{CaCO}}_{3} + {\text{H}}_{2} {\text{O}}$$

In the chronoamperometry test, cathodic polarization was applied to the steel electrode surface, which increases the local pH at the cathode as illustrated in Eq. (1), while increasing the hydroxyl ion concentration enables calcium carbonate to precipitate as given in Eq. (2). The electrochemical measurements were carried out in a cell in three-electrode mode using a platinum sheet and saturated calomel electrode (SCE) as counter and reference electrodes, respectively. The material used for constructing the working electrode was steel that had the following chemical composition (wt.%): C, 0.21; S, 0.04; Mn, 2.5; P, 0.04; Si, 0.35; balance Fe. The steel electrode was polarized to − 1.0 V (vs. SCE) in test solution in the absence and presence of different concentrations of polymer **2** for 3 h using a Gamry instrument G300™ Potentiostat/Galvanostat/ZRA.

### Electrochemical impedance spectroscopy

EIS measurements were performed at − 1.0 V (vs SCE) after the scale deposition process. The frequency range for EIS measurements was 0.1 to 1 × 10^4^ Hz with an applied potential signal amplitude of 10 mV. All measurements were performed at 40.0 ± 0.1 °C in solutions open to the atmosphere without stirring. To test the reliability and reproducibility of the measurements, triplicate experiments were performed in each case under the same conditions.

## Conclusions

We report the synthesis of poly (5-nitro-2-orthanilic acid), a new type of polyaniline containing two types of functionalities by an aniline-initiated oxidative polymerization reaction. The nitro-orthanilic acid monomer itself did not polymerize under similar reaction conditions. The obtained polymer, as synthesized without further doping, was characterized with IR and UV spectroscopic techniques, elemental composition, cyclic voltammetry, viscosity, electrical conductivity, and dielectric measurements. SEM, TEM, TGA, and DSC measurements were also investigated for additional analysis. Elemental analysis of the carbon and nitrogen present is in good agreement with the given chemical formula of a modified polyaniline backbone. Analysis of sulfur contents resulted in an S/N ratio = 0.66 indicating a high content of substituted units in the resulting polymer backbone. The observed moderate reaction yield was expected because of the presence of electron-withdrawing groups that form a stable radical intermediate and the reaction occurs slowly due to electron density decreasing on the aniline’ nitrogen atom. The presence of hydrophilic –SO_3_H and a hydrophobic –NO_2_ groups on one aromatic ring gave the structure amphiphilicity or self-assembly nature and therefore the polymer morphology has a regular flower-leaf like microstructure as judged by SEM. The electronic spectra show absorption bands at 255 nm; and 380 nm, and their bandgap energies were 4.86 eV and 3.26 eV, respectively. Because of the random substitution of the monomers in such polymerization, substituents disrupt the conjugation length, which results in a hypsochromic shift of π–π* and polaron-π* transitions. The voltammogram wave showed cathodic and anodic peaks corresponding to the quinoid and benzenoid structures, respectively, in the polymer main chain. Thermogravimetric measurements revealed high thermal stability up to 500 °C and the degradation curves showed subsequent weight-losses within four steps in which the third- (major peak) and fourth peaks from 281 to 346 °C and 345–466 °C, respectively, are likely due to decomposition and/or elimination processes of the side-chain substituents. The oxidative thermal decomposition of polymer backbones was suggested above 500 °C for the remaining polymeric residues (31%) as the final weight loss. Differential thermogravimetric analysis unambiguously pointed out the glass temperature (T_g_) at 240 °C. The electrical conductivity decreases as the temperature increases at different applied frequencies. This behavior is indicative of a semiconducting nature in the extrinsic range which is characterized by high carriers and low mobility. The presence of such electron withdrawing residues causes a decrease in conductivity and increases the steepness of the temperature dependence of conductivity. The dielectric measurements indicated that the permittivity, the measure of resistance, gradually decreased in the lower frequency range, up to 10 kHz, mainly due to the charge carrier transport causing the expected high conductivity. At a higher frequency range, up to 20 MHz, a slight effect was observed for frequency on decreasing the permittivity. Assessment of the copolymer as a new scale inhibitor of CaSO_4_, using the NACE test, indicated moderate CaSO_4_ inhibition efficiency, and this result is attributed to the change in polymer geometry via intramolecular H-bonding interactions of the –SO_3_H group and the polymer chain in an aqueous medium. On the other hand, the copolymer exerted good CaCO_3_ inhibition with increasing inhibitor concentration despite the observed anomalous behavior. Thus, the abovementioned features achieved by polymerization of (5-nitro-2-orthanilic acid) make the obtained polymer highly promising for application as a new multifunctional scaling inhibitor of CaSO_4_ and CaCO_3_ precipitation, a common problem in industry. The noteworthy, derivatives-based monomers used in this work were actively used as sensing elements of optical sensors to determine sulfate in water and soil extracts.

## Supplementary Information

Supplementary Information 1.

Supplementary Information 2.

## Data Availability

All data generated or analysed during this study are included in this published article [and its supplementary information files].
