# Supplementary material for: Assessment of self-doped poly (5-nitro-2-orthanilic acid) as a scaling inhibitor to control the precipitation of CaCO3 and CaSO4 in solution
Source: Sci Rep. 2022 Jun 13;12:9722. doi: 10.1038/s41598-022-13564-9 (PMC9192702; doi:10.1038/s41598-022-13564-9)
Supplement: Supplementary file 1 — Supplementary Information 1. [file 41598_2022_13564_MOESM1_ESM.zip › dielectric/Dr Marwa Alex P2 D-(RT).pdf]

Dr Marwa Alex P2 D: 10   T: 0.2, 15.11.2021, 10:16

Fixed value(s) : AC Volt [Vrms]=1.0000e+00

Freq. [Hz]	Eps'	Eps''	Modulus'	Modulus''	Sig' [S/cm]	Sig'' [S/cm]	Zs' [Ohms]	Zs'' [Ohms]
2.00000e+07	1.86171e+00	1.31154e-01	5.34489e-01	3.76538e-02	1.45928e-06	-9.58779e-06	8.61766e+01	-1.22326e+03
1.37931e+07	1.85278e+00	1.55003e-01	5.35979e-01	4.48400e-02	1.18941e-06	-6.54374e-06	1.48804e+02	-1.77868e+03
1.00000e+07	1.88569e+00	1.79834e-01	5.25530e-01	5.01185e-02	1.00046e-06	-4.92733e-06	2.29408e+02	-2.40552e+03
6.56034e+06	1.93929e+00	2.16991e-01	5.09277e-01	5.69840e-02	7.91948e-07	-3.42811e-06	3.97592e+02	-3.55335e+03
4.52437e+06	2.00077e+00	2.54065e-01	4.91877e-01	6.24604e-02	6.39488e-07	-2.51896e-06	6.31913e+02	-4.97633e+03
3.12025e+06	2.07466e+00	3.13215e-01	4.71265e-01	7.11477e-02	5.43704e-07	-1.86548e-06	1.04371e+03	-6.91331e+03
2.15190e+06	2.12074e+00	3.38402e-01	4.59825e-01	7.33734e-02	4.05121e-07	-1.34170e-06	1.56073e+03	-9.78097e+03
1.48407e+06	2.19845e+00	3.96435e-01	4.40540e-01	7.94401e-02	3.27307e-07	-9.89475e-07	2.45017e+03	-1.35876e+04
1.00000e+06	2.29497e+00	4.68724e-01	4.18288e-01	8.54311e-02	2.60763e-07	-7.20424e-07	3.91045e+03	-1.91463e+04
7.05859e+05	2.39529e+00	5.37626e-01	3.97463e-01	8.92113e-02	2.11119e-07	-5.47911e-07	5.78513e+03	-2.57745e+04
4.86799e+05	2.52123e+00	6.21111e-01	3.73938e-01	9.21204e-02	1.68209e-07	-4.11978e-07	8.66197e+03	-3.51609e+04
3.35724e+05	2.67101e+00	7.13515e-01	3.49453e-01	9.33505e-02	1.33265e-07	-3.12098e-07	1.27276e+04	-4.76451e+04
2.31534e+05	2.84864e+00	8.11139e-01	3.24716e-01	9.24616e-02	1.04481e-07	-2.38120e-07	1.82793e+04	-6.41950e+04
1.59678e+05	3.05364e+00	9.08228e-01	3.00863e-01	8.94842e-02	8.06807e-08	-1.82431e-07	2.56514e+04	-8.62451e+04
1.00000e+05	3.35360e+00	1.02206e+00	2.72845e-01	8.31534e-02	5.68598e-08	-1.30937e-07	3.80620e+04	-1.24890e+05
7.59469e+04	3.54715e+00	1.07984e+00	2.58006e-01	7.85433e-02	4.56245e-08	-1.07620e-07	4.73380e+04	-1.55500e+05
5.23772e+04	3.81787e+00	1.14698e+00	2.40244e-01	7.21747e-02	3.34214e-08	-8.21091e-08	6.30745e+04	-2.09952e+05
3.61222e+04	4.08884e+00	1.20374e+00	2.25062e-01	6.62573e-02	2.41899e-08	-6.20725e-08	8.39597e+04	-2.85193e+05
2.49118e+04	4.34859e+00	1.26966e+00	2.11896e-01	6.18672e-02	1.75963e-08	-4.64085e-08	1.13675e+05	-3.89339e+05
1.71806e+04	4.59945e+00	1.36194e+00	1.99891e-01	5.91895e-02	1.30174e-08	-3.44036e-08	1.57695e+05	-5.32556e+05
1.00000e+04	4.95919e+00	1.59206e+00	1.82806e-01	5.86865e-02	8.85704e-09	-2.20260e-08	2.68627e+05	-8.36759e+05
8.17150e+03	5.09633e+00	1.72036e+00	1.76147e-01	5.94619e-02	7.82080e-09	-1.86220e-08	3.33079e+05	-9.86699e+05
5.63552e+03	5.36010e+00	2.04017e+00	1.62956e-01	6.20245e-02	6.39630e-09	-1.36697e-08	5.03779e+05	-1.32357e+06
3.88656e+03	5.65519e+00	2.50077e+00	1.47906e-01	6.54052e-02	5.40716e-09	-1.00654e-08	7.70295e+05	-1.74193e+06
2.68039e+03	6.01136e+00	3.14549e+00	1.30595e-01	6.83347e-02	4.69045e-09	-7.47280e-09	1.16696e+06	-2.23018e+06
1.84855e+03	6.45256e+00	4.02614e+00	1.11549e-01	6.96017e-02	4.14045e-09	-5.60738e-09	1.72346e+06	-2.76213e+06
1.00000e+03	7.43083e+00	6.19351e+00	7.94089e-02	6.61863e-02	3.44561e-09	-3.57764e-09	3.02956e+06	-3.63480e+06
8.79213e+02	7.67683e+00	6.82268e+00	7.27781e-02	6.46806e-02	3.33717e-09	-3.26583e-09	3.36737e+06	-3.78894e+06
6.06354e+02	8.49157e+00	9.03627e+00	5.52258e-02	5.87683e-02	3.04821e-09	-2.52713e-09	4.43638e+06	-4.16895e+06
4.18175e+02	9.50511e+00	1.21183e+01	4.00721e-02	5.10889e-02	2.81922e-09	-1.97864e-09	5.59216e+06	-4.38627e+06
2.88397e+02	1.07989e+01	1.63719e+01	2.80742e-02	4.25625e-02	2.62675e-09	-1.57216e-09	6.75535e+06	-4.45583e+06
1.98894e+02	1.24882e+01	2.24181e+01	1.89638e-02	3.40428e-02	2.48056e-09	-1.27117e-09	7.83455e+06	-4.36431e+06
1.37168e+02	1.48201e+01	3.07689e+01	1.27063e-02	2.63802e-02	2.34799e-09	-1.05462e-09	8.80310e+06	-4.24009e+06
1.00000e+02	1.74674e+01	4.04470e+01	8.99884e-03	2.08375e-02	2.25017e-09	-9.16124e-10	9.53797e+06	-4.11905e+06
6.52406e+01	2.24011e+01	5.86957e+01	5.67549e-03	1.48710e-02	2.13036e-09	-7.76755e-10	1.04336e+07	-3.98196e+06
4.49935e+01	2.85727e+01	8.18107e+01	3.80493e-03	1.08944e-02	2.04781e-09	-6.90175e-10	1.10832e+07	-3.87086e+06
3.10300e+01	3.69977e+01	1.12762e+02	2.62690e-03	8.00632e-03	1.94659e-09	-6.21421e-10	1.18103e+07	-3.87501e+06
2.14000e+01	4.85866e+01	1.55603e+02	1.82843e-03	5.85570e-03	1.85251e-09	-5.66536e-10	1.25249e+07	-3.91089e+06

1.47586e+01	6.46855e+01	2.15091e+02	1.28221e-03	4.26359e-03	1.76603e-09	-5.22897e-10	1.32233e+07	-3.97672e+06
1.00000e+01	8.90265e+01	3.03926e+02	8.87632e-04	3.03027e-03	1.69082e-09	-4.89714e-10	1.38705e+07	-4.06297e+06
7.01956e+00	1.19481e+02	4.13116e+02	6.46052e-04	2.23378e-03	1.61328e-09	-4.62688e-10	1.45660e+07	-4.21278e+06
4.84108e+00	1.61710e+02	5.66731e+02	4.65574e-04	1.63166e-03	1.52633e-09	-4.32826e-10	1.54276e+07	-4.40208e+06
3.33867e+00	2.24513e+02	7.84664e+02	3.37054e-04	1.17799e-03	1.45743e-09	-4.15150e-10	1.61502e+07	-4.62101e+06
2.30253e+00	3.04997e+02	1.06629e+03	2.47967e-04	8.66906e-04	1.36587e-09	-3.89407e-10	1.72336e+07	-4.92945e+06
1.58795e+00	4.23461e+02	1.47178e+03	1.80545e-04	6.27503e-04	1.30020e-09	-3.73211e-10	1.80879e+07	-5.20426e+06
1.00000e+00	6.39000e+02	2.15985e+03	1.25954e-04	4.25731e-04	1.20158e-09	-3.54936e-10	1.94870e+07	-5.76531e+06
7.55269e-01	8.29242e+02	2.74032e+03	1.01164e-04	3.34308e-04	1.15142e-09	-3.48007e-10	2.02608e+07	-6.13106e+06
5.20876e-01	1.15965e+03	3.72021e+03	7.63693e-05	2.44997e-04	1.07803e-09	-3.35749e-10	2.15297e+07	-6.71113e+06
3.59224e-01	1.61590e+03	5.04022e+03	5.76799e-05	1.79912e-04	1.00727e-09	-3.22731e-10	2.29248e+07	-7.34970e+06
2.47741e-01	2.25543e+03	6.77621e+03	4.42207e-05	1.32856e-04	9.33929e-10	-3.10716e-10	2.45469e+07	-8.17030e+06
1.70856e-01	3.09548e+03	8.98200e+03	3.42957e-05	9.95144e-05	8.53753e-10	-2.94135e-10	2.66604e+07	-9.18800e+06
1.00000e-01	4.87375e+03	1.38949e+04	2.24782e-05	6.40845e-05	7.73009e-10	-2.71084e-10	2.93335e+07	-1.02890e+07
