# Supplementary material for: Assessment of self-doped poly (5-nitro-2-orthanilic acid) as a scaling inhibitor to control the precipitation of CaCO3 and CaSO4 in solution
Source: Sci Rep. 2022 Jun 13;12:9722. doi: 10.1038/s41598-022-13564-9 (PMC9192702; doi:10.1038/s41598-022-13564-9)
Supplement: Supplementary file 1 — Supplementary Information 1. [file 41598_2022_13564_MOESM1_ESM.zip › dielectric/Dr Marwa Alex P2 D.pdf]

Dr Marwa Alex P2 D: 10 T: 0.2, 15.11.2021, 10:22

Fixed value(s) : Temp. [°C]=4.0302e+01 AC Volt [Vrms]=1.000e+00

Freq. [Hz]	Eps' Eps''	Modulus'	Modulus''	Sig' [S/cm]	Sig'' [S/cm]	Zs' [Ohms]	Zs'' [Ohms]		
2.00000e+07	1.74584e+00	1.14187e-01	5.70349e-01	3.73036e-02	1.27050e-06	-8.29865e-06	8.53752e+01	-1.30533e+03	
1.37931e+07	1.78160e+00	1.23997e-01	5.58586e-01	3.88768e-02	9.51485e-07	-5.99760e-06	1.29015e+02	-1.85370e+03	
1.00000e+07	1.81164e+00	1.28907e-01	5.49206e-01	3.90788e-02	7.17145e-07	-4.51536e-06	1.78876e+02	-2.51389e+03	
6.56034e+06	1.85090e+00	1.49219e-01	5.36790e-01	4.32757e-02	5.44600e-07	-3.10551e-06	3.01946e+02	-3.74532e+03	
4.52437e+06	1.91059e+00	1.74479e-01	5.19069e-01	4.74023e-02	4.39167e-07	-2.29198e-06	4.79570e+02	-5.25144e+03	
3.12025e+06	1.96664e+00	2.15320e-01	5.02457e-01	5.50121e-02	3.73770e-07	-1.67798e-06	8.07011e+02	-7.37089e+03	
2.15190e+06	1.99631e+00	2.17969e-01	4.95023e-01	5.40496e-02	2.60943e-07	-1.19274e-06	1.14969e+03	-1.05297e+04	
1.48407e+06	2.04720e+00	2.53255e-01	4.81110e-01	5.95174e-02	2.09094e-07	-8.64592e-07	1.83570e+03	-1.48389e+04	
1.00000e+06	2.10889e+00	2.97198e-01	4.64948e-01	6.55234e-02	1.65339e-07	-6.16907e-07	2.99921e+03	-2.12821e+04	
7.05859e+05	2.17176e+00	3.37068e-01	4.49625e-01	6.97841e-02	1.32362e-07	-4.60135e-07	4.52532e+03	-2.91571e+04	
4.86799e+05	2.24894e+00	3.86182e-01	4.31918e-01	7.41679e-02	1.04585e-07	-3.38236e-07	6.97392e+03	-4.06128e+04	
3.35724e+05	2.33908e+00	4.41875e-01	4.12787e-01	7.79793e-02	8.25297e-08	-2.50103e-07	1.06318e+04	-5.62801e+04	
2.31534e+05	2.44489e+00	5.02834e-01	3.92418e-01	8.07075e-02	6.47691e-08	-1.86113e-07	1.59555e+04	-7.75792e+04	
1.59678e+05	2.56621e+00	5.67185e-01	3.71530e-01	8.21156e-02	5.03849e-08	-1.39132e-07	2.35391e+04	-1.06502e+05	
1.00000e+05	2.74922e+00	6.51367e-01	3.44407e-01	8.15997e-02	3.62372e-08	-9.73134e-08	3.73508e+04	-1.57646e+05	
7.59469e+04	2.87213e+00	6.98984e-01	3.28705e-01	7.99965e-02	2.95329e-08	-7.90996e-08	4.82138e+04	-1.98111e+05	
5.23772e+04	3.05522e+00	7.57756e-01	3.08342e-01	7.64750e-02	2.20801e-08	-5.98865e-08	6.68326e+04	-2.69464e+05	
3.61222e+04	3.25559e+00	8.02876e-01	2.89554e-01	7.14081e-02	1.61344e-08	-4.53277e-08	9.04867e+04	-3.66915e+05	
2.49118e+04	3.46552e+00	8.34566e-01	2.72740e-01	6.56813e-02	1.15663e-08	-3.41699e-08	1.20683e+05	-5.01134e+05	
1.71806e+04	3.67931e+00	8.51188e-01	2.57983e-01	5.96829e-02	8.13566e-09	-2.56089e-08	1.59009e+05	-6.87327e+05	
1.00000e+04	3.99005e+00	8.57940e-01	2.39548e-01	5.15075e-02	4.77294e-09	-1.66344e-08	2.35766e+05	-1.09649e+06	
8.17150e+03	4.10372e+00	8.57000e-01	2.33498e-01	4.87624e-02	3.89594e-09	-1.41096e-08	2.73146e+05	-1.30795e+06	
5.63552e+03	4.31016e+00	8.52477e-01	2.23276e-01	4.41601e-02	2.67267e-09	-1.03780e-08	3.58680e+05	-1.81350e+06	
3.88656e+03	4.51368e+00	8.43728e-01	2.14069e-01	4.00153e-02	1.82431e-09	-7.59726e-09	4.71271e+05	-2.52115e+06	
2.68039e+03	4.71304e+00	8.31795e-01	2.05768e-01	3.63156e-02	1.24035e-09	-5.53677e-09	6.20164e+05	-3.51391e+06	
1.84855e+03	4.90359e+00	8.18338e-01	1.98406e-01	3.31111e-02	8.41573e-10	-4.01443e-09	8.19888e+05	-4.91288e+06	
1.00000e+03	5.19633e+00	8.02911e-01	1.87956e-01	2.90420e-02	4.46680e-10	-2.33453e-09	1.32934e+06	-8.60334e+06	
8.79213e+02	5.25249e+00	8.02844e-01	1.86039e-01	2.84361e-02	3.92694e-10	-2.08002e-09	1.48043e+06	-9.68549e+06	
6.06354e+02	5.40604e+00	8.13888e-01	1.80879e-01	2.72316e-02	2.74549e-10	-1.48629e-09	2.05569e+06	-1.36544e+07	
4.18175e+02	5.53709e+00	8.50831e-01	1.76434e-01	2.71109e-02	1.97939e-10	-1.05552e-09	2.96754e+06	-1.93124e+07	
2.88397e+02	5.66598e+00	9.34765e-01	1.71816e-01	2.83459e-02	1.49976e-10	-7.48620e-10	4.49895e+06	-2.72699e+07	
1.98894e+02	5.77973e+00	1.08635e+00	1.67115e-01	3.14106e-02	1.20204e-10	-5.28877e-10	7.22878e+06	-3.84595e+07	
1.37168e+02	5.89636e+00	1.32434e+00	1.61451e-01	3.62623e-02	1.01060e-10	-3.73643e-10	1.21008e+07	-5.38764e+07	
1.00000e+02	6.00276e+00	1.62280e+00	1.55244e-01	4.19689e-02	9.02804e-11	-2.78317e-10	1.92105e+07	-7.10601e+07	
6.52406e+01	6.16904e+00	2.20792e+00	1.43693e-01	5.14282e-02	8.01364e-11	-1.87611e-10	3.60823e+07	-1.00816e+08	
4.49935e+01	6.34688e+00	2.94999e+00	1.29567e-01	6.02219e-02	7.38413e-11	-1.33838e-10	6.12654e+07	-1.31812e+08	
3.10300e+01	6.56253e+00	4.00304e+00	1.11058e-01	6.77435e-02	6.91037e-11	-9.60248e-11	9.99302e+07	-1.63824e+08	
2.14000e+01	6.82392e+00	5.47548e+00	8.91471e-02	7.15312e-02	6.51877e-11	-6.93360e-11	1.53000e+08	-1.90679e+08	
1.47586e+01	7.13504e+00	7.55874e+00	6.60387e-02	6.99603e-02	6.20619e-11	-5.03724e-11	2.16978e+08	-2.04816e+08	
1.00000e+01	7.54577e+00	1.06997e+01	4.40189e-02	6.24173e-02	5.95249e-11	-3.64158e-11	2.85704e+08	-2.01488e+08	
7.01956e+00	8.02670e+00	1.47453e+01	2.84784e-02	5.23157e-02	5.75829e-11	-2.74404e-11	3.41141e+08	-1.85702e+08	
4.84108e+00	8.71291e+00	2.07588e+01	1.71906e-02	4.09571e-02	5.59079e-11	-2.07725e-11	3.87256e+08	-1.62540e+08	
3.33867e+00	9.64264e+00	2.91064e+01	1.02564e-02	3.09589e-02	5.40619e-11	-1.60527e-11	4.24446e+08	-1.40615e+08	

**2.30253e+00**	**1.09695e+01**	**4.11533e+01**	**6.04738e-03**	**2.26874e-02**	**5.27157e-11**	**-1.27705e-11**	**4.51015e+08**	**-1.20219e+08**
**1.58795e+00**	**1.27687e+01**	**5.78651e+01**	**3.63635e-03**	**1.64792e-02**	**5.11191e-11**	**-1.03967e-11**	**4.75016e+08**	**-1.04819e+08**
**1.00000e+00**	**1.62037e+01**	**8.83753e+01**	**2.00721e-03**	**1.09474e-02**	**4.91654e-11**	**-8.45820e-12**	**5.01095e+08**	**-9.18762e+07**
**7.55269e-01**	**1.89453e+01**	**1.14907e+02**	**1.39688e-03**	**8.47237e-03**	**4.82812e-11**	**-7.54016e-12**	**5.13469e+08**	**-8.46578e+07**
**5.20876e-01**	**2.43044e+01**	**1.61576e+02**	**9.10365e-04**	**6.05211e-03**	**4.68208e-11**	**-6.75306e-12**	**5.31844e+08**	**-8.00005e+07**
**3.59224e-01**	**3.21830e+01**	**2.28379e+02**	**6.05027e-04**	**4.29343e-03**	**4.56406e-11**	**-6.23180e-12**	**5.47078e+08**	**-7.70939e+07**
**2.47741e-01**	**4.27670e+01**	**3.16728e+02**	**4.18685e-04**	**3.10074e-03**	**4.36530e-11**	**-5.75652e-12**	**5.72900e+08**	**-7.73572e+07**
**1.70856e-01**	**5.79160e+01**	**4.39200e+02**	**2.95112e-04**	**2.23795e-03**	**4.17466e-11**	**-5.40995e-12**	**5.99559e+08**	**-7.90621e+07**
**1.00000e-01**	**9.21140e+01**	**6.96842e+02**	**1.86438e-04**	**1.41040e-03**	**3.87671e-11**	**-5.06891e-12**	**6.45585e+08**	**-8.53383e+07**
