# Supplementary material for: Assessment of self-doped poly (5-nitro-2-orthanilic acid) as a scaling inhibitor to control the precipitation of CaCO3 and CaSO4 in solution
Source: Sci Rep. 2022 Jun 13;12:9722. doi: 10.1038/s41598-022-13564-9 (PMC9192702; doi:10.1038/s41598-022-13564-9)
Supplement: Supplementary file 1 — Supplementary Information 1. [file 41598_2022_13564_MOESM1_ESM.zip › dielectric/Dr Marwa Alex P2 D1.pdf]

Dr Marwa Alex P2 D: 10   T: 0.2, 15.11.2021, 10:22

Fixed value(s) : Temp. [°C]=6.0247e+01 AC Volt [Vrms]=1.000e+00

Freq. [Hz]	Eps'	Eps''	Modulus'	Modulus''	Sig' [S/cm]	Sig'' [S/cm]	Zs' [Ohms]	Zs'' [Ohms]	
2.00000e+07	1.69054e+00	8.17828e-02	5.90146e-01	2.85493e-02	9.09958e-07	-7.68331e-06	6.53396e+01	-1.35064e+03	
1.37931e+07	1.71633e+00	9.78901e-02	5.80750e-01	3.31228e-02	7.51155e-07	-5.49671e-06	1.09920e+02	-1.92725e+03	
1.00000e+07	1.74590e+00	9.97169e-02	5.70907e-01	3.26072e-02	5.54751e-07	-4.14966e-06	1.49253e+02	-2.61322e+03	
6.56034e+06	1.77652e+00	1.09214e-01	5.60779e-01	3.44746e-02	3.98596e-07	-2.83405e-06	2.40538e+02	-3.91270e+03	
4.52437e+06	1.82804e+00	1.30514e-01	5.44259e-01	3.88576e-02	3.28507e-07	-2.08420e-06	3.93124e+02	-5.50628e+03	
3.12025e+06	1.87357e+00	1.65331e-01	5.29617e-01	4.67353e-02	2.86994e-07	-1.51641e-06	6.85593e+02	-7.76931e+03	
2.15190e+06	1.89495e+00	1.60437e-01	5.23961e-01	4.43613e-02	1.92068e-07	-1.07140e-06	9.43611e+02	-1.11452e+04	
1.48407e+06	1.93311e+00	1.86036e-01	5.12553e-01	4.93262e-02	1.53596e-07	-7.70403e-07	1.52137e+03	-1.58087e+04	
1.00000e+06	1.97866e+00	2.17679e-01	4.99349e-01	5.49352e-02	1.21101e-07	-5.44453e-07	2.51456e+03	-2.28568e+04	
7.05859e+05	2.02446e+00	2.44872e-01	4.86836e-01	5.88860e-02	9.61583e-08	-4.02294e-07	3.81861e+03	-3.15700e+04	
4.86799e+05	2.08000e+00	2.78673e-01	4.72291e-01	6.32761e-02	7.54699e-08	-2.92485e-07	5.94978e+03	-4.44090e+04	
3.35724e+05	2.14380e+00	3.17217e-01	4.56467e-01	6.75432e-02	5.92472e-08	-2.13630e-07	9.20896e+03	-6.22355e+04	
2.31534e+05	2.21740e+00	3.59939e-01	4.39402e-01	7.13259e-02	4.63631e-08	-1.56810e-07	1.41008e+04	-8.68677e+04	
1.59678e+05	2.30021e+00	4.06092e-01	4.21603e-01	7.44322e-02	3.60744e-08	-1.15501e-07	2.13366e+04	-1.20856e+05	
1.00000e+05	2.42371e+00	4.71395e-01	3.97552e-01	7.73213e-02	2.62249e-08	-7.92046e-08	3.53924e+04	-1.81972e+05	
7.59469e+04	2.50641e+00	5.12159e-01	3.82985e-01	7.82591e-02	2.16393e-08	-6.36477e-08	4.71667e+04	-2.30825e+05	
5.23772e+04	2.63174e+00	5.69721e-01	3.62967e-01	7.85753e-02	1.66010e-08	-4.75469e-08	6.86681e+04	-3.17202e+05	
3.61222e+04	2.77438e+00	6.25268e-01	3.43018e-01	7.73067e-02	1.25652e-08	-3.56574e-08	9.79613e+04	-4.34664e+05	
2.49118e+04	2.93134e+00	6.79443e-01	3.23748e-01	7.50401e-02	9.41647e-09	-2.67666e-08	1.37879e+05	-5.94856e+05	
1.71806e+04	3.10357e+00	7.27505e-01	3.05427e-01	7.15949e-02	6.95350e-09	-2.01059e-08	1.90746e+05	-8.13730e+05	
1.00000e+04	3.37740e+00	7.85301e-01	2.80899e-01	6.53137e-02	4.36883e-09	-1.32261e-08	2.98961e+05	-1.28576e+06	
8.17150e+03	3.48323e+00	8.02369e-01	2.72624e-01	6.27995e-02	3.64758e-09	-1.12888e-08	3.51775e+05	-1.52712e+06	
5.63552e+03	3.68353e+00	8.28431e-01	2.58408e-01	5.81162e-02	2.59728e-09	-8.41338e-09	4.72035e+05	-2.09886e+06	
3.88656e+03	3.88820e+00	8.47966e-01	2.45511e-01	5.35428e-02	1.83347e-09	-6.24486e-09	6.30588e+05	-2.89146e+06	
2.68039e+03	4.09744e+00	8.61743e-01	2.33717e-01	4.91537e-02	1.28501e-09	-4.61881e-09	8.39400e+05	-3.99120e+06	
1.84855e+03	4.30900e+00	8.69351e-01	2.22996e-01	4.49899e-02	8.94035e-10	-3.40295e-09	1.11403e+06	-5.52176e+06	
1.00000e+03	4.66459e+00	8.67361e-01	2.07216e-01	3.85310e-02	4.82535e-10	-2.03871e-09	1.76368e+06	-9.48495e+06	
8.79213e+02	4.72929e+00	8.61270e-01	2.04661e-01	3.72716e-02	4.21272e-10	-1.82410e-09	1.94042e+06	-1.06549e+07	
6.06354e+02	4.93090e+00	8.49305e-01	1.96960e-01	3.39246e-02	2.86496e-10	-1.32601e-09	2.56094e+06	-1.48683e+07	
4.18175e+02	5.11594e+00	8.35306e-01	1.90392e-01	3.10862e-02	1.94327e-10	-9.57539e-10	3.40268e+06	-2.08402e+07	
2.88397e+02	5.29701e+00	8.27292e-01	1.84291e-01	2.87827e-02	1.32733e-10	-6.89422e-10	4.56828e+06	-2.92499e+07	
1.98894e+02	5.45474e+00	8.32603e-01	1.79153e-01	2.73456e-02	9.21275e-11	-4.92916e-10	6.29328e+06	-4.12299e+07	
1.37168e+02	5.59186e+00	8.65605e-01	1.74647e-01	2.70348e-02	6.60546e-11	-3.50406e-10	9.02154e+06	-5.82796e+07	
1.00000e+02	5.69349e+00	9.28276e-01	1.71091e-01	2.78950e-02	5.16424e-11	-2.61111e-10	1.27684e+07	-7.83138e+07	
6.52406e+01	5.81274e+00	1.09579e+00	1.66132e-01	3.13183e-02	3.97716e-11	-1.74679e-10	2.19731e+07	-1.16559e+08	
4.49935e+01	5.90662e+00	1.33951e+00	1.61020e-01	3.65163e-02	3.35293e-11	-1.22818e-10	3.71491e+07	-1.63811e+08	
3.10300e+01	5.99903e+00	1.72530e+00	1.53959e-01	4.42783e-02	2.97836e-11	-8.62972e-11	6.53160e+07	-2.27109e+08	
2.14000e+01	6.09676e+00	2.29845e+00	1.43611e-01	5.41405e-02	2.73639e-11	-6.06789e-11	1.15803e+08	-3.07174e+08	
1.47586e+01	6.20777e+00	3.13502e+00	1.28353e-01	6.48204e-02	2.57405e-11	-4.27590e-11	2.01037e+08	-3.98081e+08	
1.00000e+01	6.34900e+00	4.39748e+00	1.06442e-01	7.37243e-02	2.44643e-11	-2.97579e-11	3.37459e+08	-4.87218e+08	
7.01956e+00	6.50909e+00	6.03527e+00	8.26103e-02	7.65968e-02	2.35687e-11	-2.15139e-11	4.99472e+08	-5.38686e+08	
4.84108e+00	6.72827e+00	8.50394e+00	5.72197e-02	7.23207e-02	2.29029e-11	-1.54275e-11	6.83804e+08	-5.41022e+08	
3.33867e+00	7.01525e+00	1.19676e+01	3.64547e-02	6.21896e-02	2.22285e-11	-1.11727e-11	8.52619e+08	-4.99794e+08	

2.30253e+00	7.40243e+00	1.68415e+01	2.18727e-02	4.97633e-02	2.15733e-11	-8.20125e-12	9.89268e+08	-4.34818e+08
1.58795e+00	7.87575e+00	2.36545e+01	1.26709e-02	3.80565e-02	2.08968e-11	-6.07418e-12	1.09699e+09	-3.65243e+08
1.00000e+00	8.87645e+00	3.67050e+01	6.22449e-03	2.57389e-02	2.04200e-11	-4.38187e-12	1.17815e+09	-2.84915e+08
7.55269e-01	9.67965e+00	4.76232e+01	4.09865e-03	2.01651e-02	2.00101e-11	-3.64698e-12	1.22211e+09	-2.48399e+08
5.20876e-01	1.11387e+01	6.72776e+01	2.39524e-03	1.44672e-02	1.94955e-11	-2.93796e-12	1.27134e+09	-2.10488e+08
3.59224e-01	1.30843e+01	9.51519e+01	1.41834e-03	1.03145e-02	1.90157e-11	-2.41499e-12	1.31429e+09	-1.80728e+08
2.47741e-01	1.63207e+01	1.33028e+02	9.08577e-04	7.40572e-03	1.83346e-11	-2.11157e-12	1.36830e+09	-1.67871e+08
1.70856e-01	2.04185e+01	1.84088e+02	5.95202e-04	5.36618e-03	1.74978e-11	-1.84576e-12	1.43763e+09	-1.59458e+08
1.00000e-01	2.93389e+01	2.93266e+02	3.37750e-04	3.37608e-03	1.63152e-11	-1.57657e-12	1.54534e+09	-1.54599e+08
