# Supplementary material for: Assessment of self-doped poly (5-nitro-2-orthanilic acid) as a scaling inhibitor to control the precipitation of CaCO3 and CaSO4 in solution
Source: Sci Rep. 2022 Jun 13;12:9722. doi: 10.1038/s41598-022-13564-9 (PMC9192702; doi:10.1038/s41598-022-13564-9)
Supplement: Supplementary file 1 — Supplementary Information 1. [file 41598_2022_13564_MOESM1_ESM.zip › dielectric/Dr Marwa Alex P2 D2.pdf]

Dr Marwa Alex P2 D: 10 T: 0.2, 15.11.2021, 10:22
Fixed value(s) : Temp. [°C]=8.0165e+01 AC Volt [Vrms]=1.000e+00

Freq. [Hz]	Eps'	Eps''	Modulus'	Modulus''	Sig' [S/cm]	Sig'' [S/cm]	Zs' [Ohms]	Zs'' [Ohms]
2.00000e+07	1.60760e+00	4.79456e-02	6.21493e-01	1.85356e-02	5.33467e-07	-6.76045e-06	4.24217e+01	-1.42239e+03
1.37931e+07	1.62280e+00	5.79120e-02	6.15434e-01	2.19626e-02	4.44385e-07	-4.77905e-06	7.28842e+01	-2.04235e+03
1.00000e+07	1.64451e+00	5.50430e-02	6.07403e-01	2.03303e-02	3.06219e-07	-3.58558e-06	9.30580e+01	-2.78028e+03
6.56034e+06	1.66287e+00	5.67327e-02	6.00671e-01	2.04933e-02	2.07057e-07	-2.41926e-06	1.42987e+02	-4.19104e+03
4.52437e+06	1.70153e+00	7.15021e-02	5.86671e-01	2.46533e-02	1.79973e-07	-1.76576e-06	2.49418e+02	-5.93537e+03
3.12025e+06	1.73411e+00	9.82641e-02	5.74819e-01	3.25724e-02	1.70574e-07	-1.27432e-06	4.77827e+02	-8.43242e+03
2.15190e+06	1.74078e+00	8.40127e-02	5.73121e-01	2.76598e-02	1.00576e-07	-8.86827e-07	5.88352e+02	-1.21909e+04
1.48407e+06	1.76157e+00	9.88353e-02	5.65893e-01	3.17501e-02	8.16010e-08	-6.28776e-07	9.79268e+02	-1.74538e+04
1.00000e+06	1.78596e+00	1.17456e-01	5.57511e-01	3.66654e-02	6.53438e-08	-4.37251e-07	1.67829e+03	-2.55190e+04
7.05859e+05	1.81055e+00	1.31423e-01	5.49423e-01	3.98812e-02	5.16083e-08	-3.18294e-07	2.58619e+03	-3.56287e+04
4.86799e+05	1.83989e+00	1.49627e-01	5.39940e-01	4.39101e-02	4.05218e-08	-2.27458e-07	4.12881e+03	-5.07700e+04
3.35724e+05	1.87352e+00	1.70766e-01	5.29358e-01	4.82496e-02	3.18943e-08	-1.63148e-07	6.57843e+03	-7.21735e+04
2.31534e+05	1.91228e+00	1.94388e-01	5.17587e-01	5.26140e-02	2.50388e-08	-1.17509e-07	1.04016e+04	-1.02325e+05
1.59678e+05	1.95495e+00	2.19601e-01	5.05148e-01	5.67437e-02	1.95079e-08	-8.48312e-08	1.62661e+04	-1.44805e+05
1.00000e+05	2.01863e+00	2.57350e-01	4.87464e-01	6.21456e-02	1.43170e-08	-5.66688e-08	2.84460e+04	-2.23127e+05
7.59469e+04	2.06099e+00	2.82135e-01	4.76279e-01	6.51992e-02	1.19205e-08	-4.48280e-08	3.92956e+04	-2.87053e+05
5.23772e+04	2.12516e+00	3.19615e-01	4.60146e-01	6.92041e-02	9.31319e-09	-3.27857e-08	6.04785e+04	-4.02128e+05
3.61222e+04	2.19908e+00	3.59092e-01	4.42926e-01	7.23265e-02	7.21621e-09	-2.40963e-08	9.16504e+04	-5.61265e+05
2.49118e+04	2.28143e+00	4.04185e-01	4.24984e-01	7.52916e-02	5.60165e-09	-1.77594e-08	1.38341e+05	-7.80867e+05
1.71806e+04	2.37514e+00	4.53221e-01	4.06236e-01	7.75173e-02	4.33189e-09	-1.31436e-08	2.06524e+05	-1.08231e+06
1.00000e+04	2.53648e+00	5.32896e-01	3.77582e-01	7.93272e-02	2.96464e-09	-8.54781e-09	3.63106e+05	-1.72831e+06
8.17150e+03	2.60365e+00	5.64122e-01	3.66854e-01	7.94846e-02	2.56451e-09	-7.29024e-09	4.45238e+05	-2.05495e+06
5.63552e+03	2.74053e+00	6.23849e-01	3.46916e-01	7.89711e-02	1.95588e-09	-5.45690e-09	6.41424e+05	-2.81774e+06
3.88656e+03	2.89434e+00	6.83957e-01	3.27229e-01	7.73269e-02	1.47885e-09	-4.09594e-09	9.10700e+05	-3.85387e+06
2.68039e+03	3.06605e+00	7.41810e-01	3.08117e-01	7.45468e-02	1.10617e-09	-3.08083e-09	1.27304e+06	-5.26172e+06
1.84855e+03	3.25377e+00	7.94495e-01	2.90042e-01	7.08216e-02	8.17053e-10	-2.31777e-09	1.75366e+06	-7.18195e+06
1.00000e+03	3.59908e+00	8.65755e-01	2.62651e-01	6.31804e-02	4.81642e-10	-1.44594e-09	2.89196e+06	-1.20224e+07
8.79213e+02	3.66684e+00	8.75476e-01	2.58007e-01	6.16004e-02	4.28221e-10	-1.30443e-09	3.20701e+06	-1.34322e+07
6.06354e+02	3.88546e+00	9.01929e-01	2.44211e-01	5.66884e-02	3.04248e-10	-9.73354e-10	4.27936e+06	-1.84353e+07
4.18175e+02	4.10413e+00	9.19087e-01	2.32021e-01	5.19593e-02	2.13818e-10	-7.22149e-10	5.68744e+06	-2.53969e+07
2.88397e+02	4.33200e+00	9.31209e-01	2.20645e-01	4.74298e-02	1.49406e-10	-5.34595e-10	7.52788e+06	-3.50198e+07
1.98894e+02	4.55463e+00	9.36871e-01	2.10644e-01	4.33288e-02	1.03665e-10	-3.93319e-10	9.97162e+06	-4.84773e+07
1.37168e+02	4.77267e+00	9.39739e-01	2.01706e-01	3.97160e-02	7.17118e-11	-2.87894e-10	1.32532e+07	-6.73095e+07
1.00000e+02	4.95786e+00	9.43589e-01	1.94649e-01	3.70460e-02	5.24943e-11	-2.20186e-10	1.69571e+07	-8.90971e+07
6.52406e+01	5.18131e+00	9.54860e-01	1.86662e-01	3.43998e-02	3.46567e-11	-1.51761e-10	2.41351e+07	-1.30963e+08
4.49935e+01	5.36214e+00	9.80535e-01	1.80458e-01	3.29991e-02	2.45438e-11	-1.09189e-10	3.35709e+07	-1.83585e+08
3.10300e+01	5.53898e+00	1.03633e+00	1.74432e-01	3.26359e-02	1.78900e-11	-7.83556e-11	4.81420e+07	-2.57310e+08
2.14000e+01	5.68993e+00	1.13707e+00	1.69000e-01	3.37727e-02	1.35372e-11	-5.58354e-11	7.22375e+07	-3.61479e+08
1.47586e+01	5.82176e+00	1.31261e+00	1.63460e-01	3.68547e-02	1.07774e-11	-3.95896e-11	1.14303e+08	-5.06962e+08
1.00000e+01	5.94359e+00	1.62242e+00	1.56581e-01	4.27419e-02	9.02592e-12	-2.75024e-11	1.95643e+08	-7.16723e+08
7.01956e+00	6.04960e+00	2.05449e+00	1.48207e-01	5.03322e-02	8.02313e-12	-1.97195e-11	3.28207e+08	-9.66428e+08
4.84108e+00	6.16774e+00	2.73345e+00	1.35517e-01	6.00590e-02	7.36176e-12	-1.39178e-11	5.67867e+08	-1.28133e+09
3.33867e+00	6.30841e+00	3.70891e+00	1.17800e-01	6.92581e-02	6.88890e-12	-9.85978e-12	9.49528e+08	-1.61503e+09

2.30253e+00	6.48008e+00	5.10461e+00	9.52274e-02	7.50143e-02	6.53880e-12	-7.01976e-12	1.49125e+09	-1.89307e+09
1.58795e+00	6.69822e+00	7.06778e+00	7.06417e-02	7.45392e-02	6.24382e-12	-5.03392e-12	2.14861e+09	-2.03626e+09
1.00000e+00	7.06618e+00	1.07299e+01	4.28095e-02	6.50055e-02	5.96930e-12	-3.37477e-12	2.97551e+09	-1.95953e+09
7.55269e-01	7.34157e+00	1.38252e+01	2.99612e-02	5.64213e-02	5.80903e-12	-2.66457e-12	3.41942e+09	-1.81580e+09
5.20876e-01	7.82816e+00	1.93476e+01	1.79706e-02	4.44150e-02	5.60648e-12	-1.97864e-12	3.90307e+09	-1.57921e+09
3.59224e-01	8.46169e+00	2.69975e+01	1.05710e-02	3.37273e-02	5.39533e-12	-1.49119e-12	4.29760e+09	-1.34698e+09
2.47741e-01	9.34519e+00	3.74555e+01	6.27089e-03	2.51337e-02	5.16230e-12	-1.15017e-12	4.64376e+09	-1.15862e+09
1.70856e-01	1.04830e+01	5.09926e+01	3.86808e-03	1.88155e-02	4.84692e-12	-9.01375e-13	5.04077e+09	-1.03628e+09
1.00000e-01	1.27405e+01	8.02354e+01	1.93036e-03	1.21568e-02	4.46370e-12	-6.53152e-13	5.56456e+09	-8.83588e+08
