# Supplementary material for: Assessment of self-doped poly (5-nitro-2-orthanilic acid) as a scaling inhibitor to control the precipitation of CaCO3 and CaSO4 in solution
Source: Sci Rep. 2022 Jun 13;12:9722. doi: 10.1038/s41598-022-13564-9 (PMC9192702; doi:10.1038/s41598-022-13564-9)
Supplement: Supplementary file 1 — Supplementary Information 1. [file 41598_2022_13564_MOESM1_ESM.zip › dielectric/Dr Marwa Alex P2 D3.pdf]

Dr Marwa Alex P2 D: 10   T: 0.2, 15.11.2021, 10:22
Fixed value(s) : Temp. [°C]=1.0011e+02 AC Volt [Vrms]=1.000e+00

Freq. [Hz]	Eps'	Eps''	Modulus'	Modulus''	Sig' [S/cm]	Sig'' [S/cm]	Zs' [Ohms]	Zs'' [Ohms]
2.00000e+07	1.56029e+00	2.84303e-02	6.40692e-01	1.16741e-02	3.16331e-07	-6.23413e-06	2.67181e+01	-1.46632e+03
1.37931e+07	1.56553e+00	3.51784e-02	6.38441e-01	1.43462e-02	2.69940e-07	-4.33954e-06	4.76086e+01	-2.11870e+03
1.00000e+07	1.58291e+00	3.03410e-02	6.31515e-01	1.21048e-02	1.68795e-07	-3.24289e-06	5.54073e+01	-2.89064e+03
6.56034e+06	1.59498e+00	2.77136e-02	6.26776e-01	1.08905e-02	1.01146e-07	-2.17150e-06	7.59860e+01	-4.37318e+03
4.52437e+06	1.62516e+00	3.99155e-02	6.14954e-01	1.51039e-02	1.00468e-07	-1.57353e-06	1.52807e+02	-6.22151e+03
3.12025e+06	1.65055e+00	6.29029e-02	6.04980e-01	2.30559e-02	1.09192e-07	-1.12928e-06	3.38223e+02	-8.87487e+03
2.15190e+06	1.65016e+00	4.45804e-02	6.05560e-01	1.63597e-02	5.33697e-08	-7.78342e-07	3.47988e+02	-1.28809e+04
1.48407e+06	1.66216e+00	5.44125e-02	6.00982e-01	1.96737e-02	4.49244e-08	-5.46698e-07	6.06798e+02	-1.85361e+04
1.00000e+06	1.67583e+00	6.70281e-02	5.95768e-01	2.38290e-02	3.72895e-08	-3.75979e-07	1.09073e+03	-2.72702e+04
7.05859e+05	1.68985e+00	7.52072e-02	5.90598e-01	2.62847e-02	2.95329e-08	-2.70897e-07	1.70449e+03	-3.82988e+04
4.86799e+05	1.70659e+00	8.62689e-02	5.84471e-01	2.95453e-02	2.33633e-08	-1.91358e-07	2.77811e+03	-5.49571e+04
3.35724e+05	1.72586e+00	9.93939e-02	5.77506e-01	3.32591e-02	1.85640e-08	-1.35570e-07	4.53461e+03	-7.87382e+04
2.31534e+05	1.74840e+00	1.14111e-01	5.69525e-01	3.71705e-02	1.46984e-08	-9.64001e-08	7.34844e+03	-1.12593e+05
1.59678e+05	1.77256e+00	1.29407e-01	5.61164e-01	4.09681e-02	1.14956e-08	-6.86291e-08	1.17439e+04	-1.60863e+05
1.00000e+05	1.80957e+00	1.53500e-01	5.48670e-01	4.65419e-02	8.53959e-09	-4.50384e-08	2.13037e+04	-2.51143e+05
7.59469e+04	1.83464e+00	1.69610e-01	5.40446e-01	4.99635e-02	7.16624e-09	-3.52646e-08	3.01130e+04	-3.25727e+05
5.23772e+04	1.87257e+00	1.94351e-01	5.28335e-01	5.48351e-02	5.66315e-09	-2.54256e-08	4.79212e+04	-4.61719e+05
3.61222e+04	1.91706e+00	2.19802e-01	5.14864e-01	5.90323e-02	4.41708e-09	-1.84289e-08	7.48043e+04	-6.52424e+05
2.49118e+04	1.96660e+00	2.50060e-01	5.00402e-01	6.36280e-02	3.46561e-09	-1.33962e-08	1.16910e+05	-9.19442e+05
1.71806e+04	2.02308e+00	2.83659e-01	4.84766e-01	6.79700e-02	2.71122e-09	-9.77857e-09	1.81088e+05	-1.29153e+06
1.00000e+04	2.12014e+00	3.41015e-01	4.59773e-01	7.39524e-02	1.89715e-09	-6.23162e-09	3.38504e+05	-2.10452e+06
8.17150e+03	2.16099e+00	3.64844e-01	4.49926e-01	7.59620e-02	1.65859e-09	-5.27788e-09	4.25505e+05	-2.52029e+06
5.63552e+03	2.24412e+00	4.13466e-01	4.30979e-01	7.94054e-02	1.29629e-09	-3.90054e-09	6.44952e+05	-3.50053e+06
3.88656e+03	2.33845e+00	4.67834e-01	4.11177e-01	8.22608e-02	1.01155e-09	-2.89399e-09	9.68809e+05	-4.84254e+06
2.68039e+03	2.44740e+00	5.28850e-01	3.90369e-01	8.43535e-02	7.88605e-10	-2.15832e-09	1.44051e+06	-6.66636e+06
1.84855e+03	2.57212e+00	5.96014e-01	3.68973e-01	8.54987e-02	6.12937e-10	-1.61676e-09	2.11710e+06	-9.13640e+06
1.00000e+03	2.82585e+00	7.19130e-01	3.32353e-01	8.45781e-02	4.00070e-10	-1.01576e-09	3.87141e+06	-1.52128e+07
8.79213e+02	2.88080e+00	7.43707e-01	3.25436e-01	8.40145e-02	3.63768e-10	-9.19955e-10	4.37392e+06	-1.69427e+07
6.06354e+02	3.06955e+00	8.18079e-01	3.04175e-01	8.10670e-02	2.75963e-10	-6.98122e-10	6.11968e+06	-2.29619e+07
4.18175e+02	3.28272e+00	8.86345e-01	2.83926e-01	7.66610e-02	2.06201e-10	-5.31056e-10	8.39126e+06	-3.10784e+07
2.88397e+02	3.51408e+00	9.41916e-01	2.65495e-01	7.11633e-02	1.51123e-10	-4.03365e-10	1.12948e+07	-4.21383e+07
1.98894e+02	3.76085e+00	9.81216e-01	2.48951e-01	6.49520e-02	1.08571e-10	-3.05488e-10	1.49480e+07	-5.72932e+07
1.37168e+02	4.01272e+00	1.00304e+00	2.34552e-01	5.86297e-02	7.65420e-11	-2.29902e-10	1.95647e+07	-7.82702e+07
1.00000e+02	4.22970e+00	1.01113e+00	2.23643e-01	5.34627e-02	5.62516e-11	-1.79677e-10	2.44716e+07	-1.02368e+08
6.52406e+01	4.49890e+00	1.01054e+00	2.11601e-01	4.75295e-02	3.66775e-11	-1.26993e-10	3.33469e+07	-1.48460e+08
4.49935e+01	4.71987e+00	1.01139e+00	2.02569e-01	4.34073e-02	2.53162e-11	-9.31122e-11	4.41595e+07	-2.06079e+08
3.10300e+01	4.93312e+00	1.02367e+00	1.94343e-01	4.03280e-02	1.76714e-11	-6.78967e-11	5.94889e+07	-2.86680e+08
2.14000e+01	5.13998e+00	1.05664e+00	1.86665e-01	3.83731e-02	1.25797e-11	-4.92880e-11	8.20775e+07	-3.99263e+08
1.47586e+01	5.32925e+00	1.11975e+00	1.79710e-01	3.77597e-02	9.19385e-12	-3.55457e-11	1.17110e+08	-5.57361e+08
1.00000e+01	5.51424e+00	1.23641e+00	1.72668e-01	3.87159e-02	6.87849e-12	-2.51139e-11	1.77215e+08	-7.90354e+08
7.01956e+00	5.67055e+00	1.40891e+00	1.66096e-01	4.12685e-02	5.50204e-12	-1.82393e-11	2.69104e+08	-1.08308e+09
4.84108e+00	5.82246e+00	1.69182e+00	1.58377e-01	4.60192e-02	4.55643e-12	-1.29879e-11	4.35119e+08	-1.49748e+09
3.33867e+00	5.96142e+00	2.11743e+00	1.48953e-01	5.29066e-02	3.93290e-12	-9.21530e-12	7.25349e+08	-2.04215e+09

2.30253e+00	6.10096e+00	2.76548e+00	1.35971e-01	6.16338e-02	3.54246e-12	-6.53411e-12	1.22525e+09	-2.70303e+09
1.58795e+00	6.25148e+00	3.71429e+00	1.18227e-01	7.02441e-02	3.28127e-12	-4.63926e-12	2.02480e+09	-3.40792e+09
1.00000e+00	6.48462e+00	5.50596e+00	8.96089e-02	7.60851e-02	3.06311e-12	-3.05123e-12	3.48265e+09	-4.10168e+09
7.55269e-01	6.65528e+00	7.03890e+00	7.09224e-02	7.50105e-02	2.95758e-12	-2.37621e-12	4.54602e+09	-4.29826e+09
5.20876e-01	6.92824e+00	9.80466e+00	4.80688e-02	6.80256e-02	2.84116e-12	-1.71786e-12	5.97791e+09	-4.22416e+09
3.59224e-01	7.26334e+00	1.36909e+01	3.02393e-02	5.69988e-02	2.73606e-12	-1.25170e-12	7.26291e+09	-3.85315e+09
2.47741e-01	7.70778e+00	1.91951e+01	1.80146e-02	4.48628e-02	2.64556e-12	-9.24498e-13	8.28895e+09	-3.32842e+09
1.70856e-01	8.18047e+00	2.57426e+01	1.12122e-02	3.52831e-02	2.44688e-12	-6.82515e-13	9.45252e+09	-3.00382e+09
1.00000e-01	9.31029e+00	4.15456e+01	5.13610e-03	2.29190e-02	2.31129e-12	-4.62323e-13	1.04907e+10	-2.35096e+09
