# Supplementary material for: Assessment of self-doped poly (5-nitro-2-orthanilic acid) as a scaling inhibitor to control the precipitation of CaCO3 and CaSO4 in solution
Source: Sci Rep. 2022 Jun 13;12:9722. doi: 10.1038/s41598-022-13564-9 (PMC9192702; doi:10.1038/s41598-022-13564-9)
Supplement: Supplementary file 2 — Supplementary Information 2. [file 41598_2022_13564_MOESM2_ESM.zip › scale inhibition/chronoamerometry, figure 9.pdf]

--	--	--	--	--	--	--	--	--	--
--	blank	--	25 ppm	--	50 ppm	--	100 ppm	--	150 ppm
0	1064	0	902.5	0	786	0	907.7	0	971.5
1.67E-04	1063	1.67E-04	896.5	1.67E-04	775.4	1.67E-04	904.8	1.67E-04	965.9
3.33E-04	1062	3.33E-04	891.2	3.33E-04	765.2	3.33E-04	902.3	3.33E-04	960.9
5.00E-04	1062	5.00E-04	886.6	5.00E-04	755.8	5.00E-04	900.2	5.00E-04	956.3
6.67E-04	1061	6.67E-04	882.7	6.67E-04	747	6.67E-04	898.5	6.67E-04	952.2
8.33E-04	1061	8.33E-04	879.5	8.33E-04	739.2	8.33E-04	897.2	8.33E-04	948.7
1.00E-03	1061	1.00E-03	877.1	1.00E-03	732.2	1.00E-03	896.1	1.00E-03	945.7
0.00117	1061	0.00117	875.3	0.00117	726.1	0.00117	895.2	0.00117	943.1
0.00133	1062	0.00133	874.2	0.00133	720.9	0.00133	894.6	0.00133	941
0.0015	1062	0.0015	873.7	0.0015	716.6	0.0015	894.1	0.0015	939.3
0.00167	1062	0.00167	873.8	0.00167	713.1	0.00167	893.7	0.00167	937.9
0.00183	1062	0.00183	874.4	0.00183	710.5	0.00183	893.4	0.00183	936.9
0.002	1061	0.002	875.4	0.002	708.5	0.002	893.1	0.002	936.1
0.00217	1061	0.00217	876.8	0.00217	707.3	0.00217	892.8	0.00217	935.5
0.00233	1061	0.00233	878.4	0.00233	706.5	0.00233	892.5	0.00233	935
0.0025	1060	0.0025	880.3	0.0025	706.2	0.0025	892.2	0.0025	934.7
0.00267	1059	0.00267	882.2	0.00267	706.3	0.00267	891.8	0.00267	934.4
0.00283	1058	0.00283	884.3	0.00283	706.7	0.00283	891.3	0.00283	934.2
0.003	1057	0.003	886.4	0.003	707.2	0.003	890.7	0.003	933.9
0.00317	1056	0.00317	888.4	0.00317	707.8	0.00317	890.1	0.00317	933.6
0.00333	1055	0.00333	890.4	0.00333	708.4	0.00333	889.4	0.00333	933.2
0.0035	1054	0.0035	892.3	0.0035	709	0.0035	888.6	0.0035	932.7
0.00367	1053	0.00367	894	0.00367	709.4	0.00367	887.7	0.00367	932.1
0.00383	1052	0.00383	895.5	0.00383	709.7	0.00383	886.8	0.00383	931.3
0.004	1051	0.004	896.9	0.004	709.9	0.004	885.8	0.004	930.5
0.00417	1050	0.00417	898.1	0.00417	709.8	0.00417	884.8	0.00417	929.5
0.00433	1049	0.00433	899.1	0.00433	709.6	0.00433	883.9	0.00433	928.4
0.0045	1048	0.0045	899.9	0.0045	709.2	0.0045	882.9	0.0045	927.3
0.00467	1047	0.00467	900.5	0.00467	708.7	0.00467	882	0.00467	926
0.00483	1047	0.00483	900.9	0.00483	708	0.00483	881.1	0.00483	924.7
0.005	1046	0.005	901.2	0.005	707.3	0.005	880.2	0.005	923.3
0.00517	1045	0.00517	901.2	0.00517	706.6	0.00517	879.4	0.00517	921.9
0.00533	1045	0.00533	901.1	0.00533	705.9	0.00533	878.6	0.00533	920.5
0.0055	1044	0.0055	900.9	0.0055	705.3	0.0055	877.9	0.0055	919.2
0.00567	1044	0.00567	900.5	0.00567	704.8	0.00567	877.3	0.00567	917.9
0.00583	1043	0.00583	899.9	0.00583	704.5	0.00583	876.6	0.00583	916.7
0.006	1043	0.006	899.1	0.006	704.5	0.006	876	0.006	915.6
0.00617	1043	0.00617	898.3	0.00617	704.6	0.00617	875.3	0.00617	914.6
0.00633	1042	0.00633	897.2	0.00633	705	0.00633	874.7	0.00633	913.7
0.0065	1042	0.0065	896	0.0065	705.5	0.0065	874	0.0065	912.9
0.00667	1041	0.00667	894.5	0.00667	706.2	0.00667	873.2	0.00667	912.2
0.00683	1041	0.00683	892.9	0.00683	707	0.00683	872.4	0.00683	911.6
0.007	1040	0.007	891	0.007	707.8	0.007	871.5	0.007	911
0.00717	1040	0.00717	888.9	0.00717	708.5	0.00717	870.5	0.00717	910.5
0.00733	1039	0.00733	886.5	0.00733	709	0.00733	869.5	0.00733	910

0.0075	1038	0.0075	883.9	0.0075	709.1	0.0075	868.5	0.0075	909.5
0.00767	1037	0.00767	881	0.00767	708.8	0.00767	867.5	0.00767	908.9
0.00783	1037	0.00783	877.8	0.00783	708	0.00783	866.5	0.00783	908.2
0.008	1036	0.008	874.4	0.008	706.6	0.008	865.5	0.008	907.4
0.00817	1036	0.00817	870.7	0.00817	704.5	0.00817	864.7	0.00817	906.4
0.00833	1035	0.00833	866.8	0.00833	701.7	0.00833	864	0.00833	905.3
0.0085	1035	0.0085	862.7	0.0085	698.2	0.0085	863.4	0.0085	904.1
0.00867	1036	0.00867	858.4	0.00867	693.9	0.00867	863	0.00867	902.6
0.00883	1036	0.00883	853.9	0.00883	689	0.00883	862.8	0.00883	901
0.009	1037	0.009	849.4	0.009	683.5	0.009	862.6	0.009	899.2
0.00917	1039	0.00917	844.8	0.00917	677.6	0.00917	862.6	0.00917	897.2
0.00933	1041	0.00933	840.2	0.00933	671.4	0.00933	862.6	0.00933	895
0.0095	1043	0.0095	835.6	0.0095	665.1	0.0095	862.5	0.0095	892.7
0.00967	1045	0.00967	830.9	0.00967	658.7	0.00967	862.3	0.00967	890.1
0.00983	1046	0.00983	826.2	0.00983	652.6	0.00983	861.7	0.00983	887.2
0.01	1048	0.01	821.5	0.01	646.7	0.01	860.6	0.01	884
0.01017	1049	0.01017	816.6	0.01017	641.3	0.01017	858.9	0.01017	880.5
0.01183	1049	0.01183	811.5	0.01183	636.4	0.01183	856.3	0.01183	876.5
0.0135	1049	0.0135	806.2	0.0135	632	0.0135	852.8	0.0135	872
0.01517	1046	0.01517	800.4	0.01517	628.2	0.01517	848	0.01517	866.9
0.01683	1042	0.01683	794.1	0.01683	624.8	0.01683	841.9	0.01683	861
0.0185	1035	0.0185	787.1	0.0185	621.7	0.0185	834.3	0.0185	854.3
0.02017	1027	0.02017	779.4	0.02017	618.9	0.02017	825.1	0.02017	846.5
0.02183	1015	0.02183	770.6	0.02183	616.1	0.02183	814.2	0.02183	837.7
0.0235	1000	0.0235	760.8	0.0235	613.1	0.0235	801.6	0.0235	827.6
0.02517	982.5	0.02517	749.8	0.02517	609.7	0.02517	787.3	0.02517	816.3
0.02683	961.5	0.02683	737.4	0.02683	605.6	0.02683	771.3	0.02683	803.5
0.0435	937.2	0.0435	723.8	0.0435	600.6	0.0435	753.8	0.0435	789.3
0.06017	909.8	0.06017	708.8	0.06017	594.5	0.06017	734.9	0.06017	773.6
0.07683	879.4	0.07683	692.4	0.07683	587.2	0.07683	714.7	0.07683	756.4
0.0935	846.3	0.0935	674.8	0.0935	578.5	0.0935	693.5	0.0935	737.9
0.11017	810.7	0.11017	655.9	0.11017	568.3	0.11017	671.5	0.11017	718
0.12683	773	0.12683	636	0.12683	556.7	0.12683	648.9	0.12683	697
0.1435	733.7	0.1435	615.2	0.1435	543.7	0.1435	626.1	0.1435	674.9
0.16017	693.2	0.16017	593.6	0.16017	529.3	0.16017	603.2	0.16017	651.9
0.17683	652.1	0.17683	571.6	0.17683	513.8	0.17683	580.5	0.17683	628.3
0.1935	610.9	0.1935	549.2	0.1935	497.3	0.1935	558.2	0.1935	604.2
0.36017	570	0.36017	526.7	0.36017	480.1	0.36017	536.5	0.36017	579.9
0.52683	529.9	0.52683	504.2	0.52683	462.4	0.52683	515.6	0.52683	555.5
0.6935	491.2	0.6935	482.1	0.6935	444.5	0.6935	495.5	0.6935	531.4
0.86017	454	0.86017	460.4	0.86017	426.6	0.86017	476.3	0.86017	507.6
1.02683	418.9	1.02683	439.2	1.02683	409	1.02683	458	1.02683	484.4
1.1935	385.9	1.1935	418.6	1.1935	391.9	1.1935	440.6	1.1935	461.9
1.36017	355.3	1.36017	398.7	1.36017	375.4	1.36017	424.1	1.36017	440.3
1.52683	327.1	1.52683	379.5	1.52683	359.6	1.52683	408.3	1.52683	419.5
1.69333	301.4	1.69333	361	1.69333	344.5	1.69333	393.2	1.69333	399.7
1.86	278.2	1.86	343.1	1.86	330.3	1.86	378.6	1.86	380.9

3.52667	257.2	3.52667	325.8	3.52667	316.8	3.52667	364.4	3.52667	363
5.19333	238.4	5.19333	308.9	5.19333	304	5.19333	350.5	5.19333	346.1
6.86	221.4	6.86	292.3	6.86	291.6	6.86	336.7	6.86	330.1
8.52667	206.2	8.52667	276	8.52667	279.7	8.52667	322.9	8.52667	314.8
10.19333	192.5	10.19333	259.9	10.19333	268	10.19333	309.1	10.19333	300.3
11.86	179.9	11.86	243.8	11.86	256.3	11.86	295.2	11.86	286.5
13.52667	168.2	13.52667	227.8	13.52667	244.5	13.52667	281.2	13.52667	273.2
15.19333	157.3	15.19333	211.7	15.19333	232.5	15.19333	267	15.19333	260.4
16.86667	146.9	16.86667	195.7	16.86667	220.2	16.86667	252.7	16.86667	248
18.53333	136.7	18.53333	179.6	18.53333	207.4	18.53333	238.3	18.53333	236
35.2	126.7	35.2	163.7	35.2	194.3	35.2	223.9	35.2	224.3
51.86667	116.8	51.86667	147.9	51.86667	180.8	51.86667	209.5	51.86667	212.9
68.53333	106.8	68.53333	132.3	68.53333	166.9	68.53333	195.4	68.53333	201.7
85.2	96.7	85.2	117	85.2	152.8	85.2	181.4	85.2	190.9
101.8667	86.45	101.8667	102.1	101.8667	138.6	101.8667	167.8	101.8667	180.3
118.5333	76.04	118.5333	87.84	118.5333	124.4	118.5333	154.7	118.5333	169.9
135.2	65.5	135.2	74.16	135.2	110.3	135.2	142	135.2	159.9
151.8667	54.83	151.8667	61.18	151.8667	96.56	151.8667	129.9	151.8667	150.2
168.5	44.09	168.5	48.96	168.5	83.22	168.5	118.3	168.5	140.8
--	--	--	--	--	--	--	--	--	--
		--							
		--							
		--							
		--							
		--							
		--							
		--							
		--							
		--							
		--							
		--							
		--							
		--							
		--							
		--							
		--							
		--							
		--							
		--							
		--							
		--							
		--							
		--							
		--							
		--							
		--							
		--							

--
--
--
--
--
--
--
--
--
--
--
--
--
--
--
--
--
--
--
--
--
--
--
--
--
--
--
--
--
--
--
--
--
--
--
--
--
--
--
--
--
--
--
--
--
--
--

--
--
--
--
--
--
--
--
--
--
--
--
--
--
--
--
--
--
--
--
--
--
--
--
--
--
--
--
--
--
--
--
--
--
--
--
--
--
--
