# Supplementary material for: Assessment of self-doped poly (5-nitro-2-orthanilic acid) as a scaling inhibitor to control the precipitation of CaCO3 and CaSO4 in solution
Source: Sci Rep. 2022 Jun 13;12:9722. doi: 10.1038/s41598-022-13564-9 (PMC9192702; doi:10.1038/s41598-022-13564-9)

concentratior	copolymer2	oxidation product
25	21.8	
50	36.7	53.3
100	62.3	73.5
150	77.2	81.9
200	36.3	85

To resize chart data range, drag lower right corner of range.

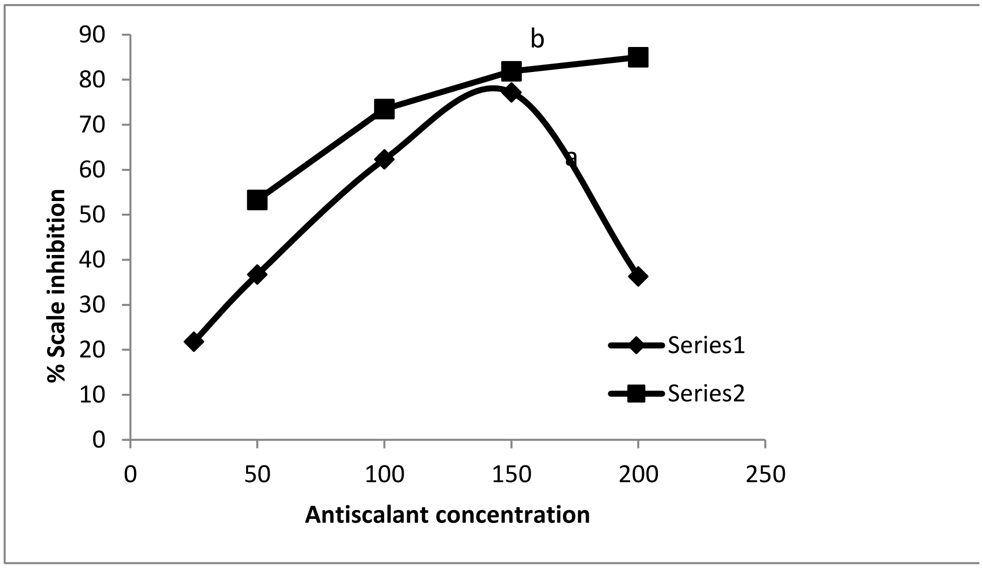

Supplement: Supplementary file 2 — Supplementary Information 2. [file 41598_2022_13564_MOESM2_ESM.zip › scale inhibition/Figure 14.pdf]
