# Supplementary material for: Assessment of self-doped poly (5-nitro-2-orthanilic acid) as a scaling inhibitor to control the precipitation of CaCO3 and CaSO4 in solution
Source: Sci Rep. 2022 Jun 13;12:9722. doi: 10.1038/s41598-022-13564-9 (PMC9192702; doi:10.1038/s41598-022-13564-9)
Supplement: Supplementary file 2 — Supplementary Information 2. [file 41598_2022_13564_MOESM2_ESM.zip › scale inhibition/impedance figure 11.pdf]

XY		XY		XY		XY		XY	
--	ZCURVE. (c	--	ZCURVE. (2	--	ZCURVE. (5	--	ZCURVE. (1	--	ZCURVE. (1
5.303	0.08767	16.21	3.704	7.006	0.4558	5.491	0.3275	4.358	-0.00447
5.333	0.1745	16.57	3.875	7.15	0.6882	5.524	0.3959	4.382	0.06705
5.36	0.2326	17.12	4.175	7.263	0.7035	5.593	0.4825	4.399	0.1053
5.4	0.2782	17.53	4.529	7.389	0.7078	5.672	0.5025	4.448	0.1524
5.439	0.3777	18.08	5.025	7.455	0.9545	5.712	0.6472	4.483	0.193
5.491	0.4055	18.61	5.635	7.567	1.088	5.812	0.8051	4.468	0.2691
5.508	0.488	19.22	6.225	7.68	1.378	5.847	0.8682	4.482	0.3497
5.567	0.5776	19.89	7.064	7.82	1.486	5.959	1.072	4.54	0.4024
5.644	0.6798	20.6	8.017	7.921	1.714	6.029	1.179	4.584	0.4549
5.667	0.7518	21.64	9.326	8.112	1.973	6.163	1.41	4.614	0.5724
5.742	0.9267	22.57	10.89	8.431	2.41	6.349	1.668	4.652	0.63
5.855	1.095	23.54	12.38	8.513	2.71	6.417	1.875	4.791	0.7833
5.942	1.279	24.27	14.23	8.725	3.12	6.627	2.169	4.78	0.8574
5.966	1.503	25.03	16.47	8.994	3.674	6.773	2.518	4.895	1.046
6.181	1.771	27.27	19.18	9.301	4.343	7.004	2.977	4.978	1.242
6.295	2.106	8.428	34.85	9.585	5.041	7.217	3.435	5.069	1.451
6.455	2.476	7.772	44.4	9.969	6.017	7.435	4.033	5.178	1.727
6.642	2.936	7.182	57.88	10.48	7.003	7.792	4.836	5.329	1.99
6.869	3.531	7.481	75.92	11.04	8.293	7.979	5.783	5.527	2.415
7.095	4.182	10.36	96.2	11.63	9.784	8.67	6.728	5.682	2.779
7.299	5.002	16.46	128.1	12.33	11.48	9.05	8.05	5.942	3.288
7.674	5.947	28.99	162	13.82	13.79	9.689	9.489	6.201	3.89
7.937	7.129	36.3	220.9	14.34	16.46	10.31	11.31	6.485	4.56
3.092	9.242	77.98	240	20.06	19.71	0.576	2.774	0.5453	1.408
3.441	11.08	119.8	279.6	22.66	23.08	0.3491	3.533	0.4422	1.798
1.78	12.48	126.5	274.1	24.69	26.95	-0.1464	4.747	0.2495	2.34
1.547	14.7	210.7	356.5	27	31.58	-0.868	6.402	-0.0103	3.142
1.869	17.44	287.4	388.4	29.87	36.68	-1.855	8.752	-0.3888	4.167
1.634	20.8	354.5	433.2	33.68	43.42	-3.08	12.15	-0.849	5.644
3.027	25.28	402.1	450.6	38.23	51.27	-4.444	16.93	-1.37	7.681
5.665	29.74	429.7	467.3	41.19	51.94	-5.074	22.96	-1.826	10.49
9.981	36.88	551.7	501.8	49.77	69.52	-5.921	32.46	-1.901	14.48
13.75	43.74	706.8	524.5	57.85	80.68	-4.602	44.17	-1.47	19.31
14.71	53.82	911.4	493.1	55.85	77.78	0.6875	57.67	-0.1207	24.59
21.06	61.74	--	--	78.82	107.5	6.745	73.45	2.294	31.67
26.7	73.58	--	--	94.32	123.4	17.27	90.03	5.9	39.21
33.18	87.1	--	--	111.2	139.5	31.49	106.1	11.2	47.14
41.28	103.3	--	--	130.9	153.9	47.82	119.8	17.38	55.42
50.93	120.8	--	--	153.6	176.4	67.44	135.6	25	64.29
66.62	142.8	--	--	178.1	198.8	89.86	150.4	33.48	73.96
80.94	166.9	--	--	209.9	218.9	114.9	164.5	43.89	84.39
99.74	196.3	--	--	245.7	236.8	142.8	178.8	56.02	95.69
122.1	229.2	--	--	291.6	251.9	168.8	185.6	70.07	107.7
149	265.7	--	--	323.9	282.3	202.5	202	84.9	120.3
186.8	298.2	--	--	371.8	302.2	237.4	212.5	107.3	131.7

218.8	355	--	--	470.4	333	272.1	219.2	125.7	144.4
282.1	392.9	--	--	529.9	336.7	326.1	221.3	160.2	149.8
340.9	453.8	--	--	581.2	336.4	357.2	214.9	189.2	158
421.2	504.4	--	--	627.7	353.1	392.7	224.3	217.9	161.5
504.6	550.4	--	--	704.9	343.3	433.2	224.4	249.9	168.6
601	592.6	--	--	758.5	351.9	462	210.1	282	163
715.1	630.3	--	--	817.4	342.3	505.6	207.4	313.1	157.2
831.2	659	--	--	882.5	328.6	526.8	199.3	341.1	151.5
951	682	--	--	925.9	334.3	566.6	198	372.2	139.1
1091	696.3	--	--	995.7	325.2	593.5	180.4	391.2	129.7
1224	694.6	--	--	1134	308.5	634.4	177.3	414.4	119.2
1352	679.1	--	--	1206	313	664.5	169.5	432.4	107.4
1479	658	--	--	--	--	--	--	445.3	97.69
1593	625.7	--	--	--	--	--	--	461.3	91.11
1696	600.1	--	--	--	--	--	--	479.5	86.85
1799	564.2	--	--	--	--	--	--	496.6	84.41
--	--	--	--	--	--	--	--	--	--

150 ppm, p6 new imp2)
